# Fourteen new species of the spider genus *Psiloderces* Simon, 1892 from Southeast Asia (Araneae, Psilodercidae)

**DOI:** 10.3897/zookeys.902.38967

**Published:** 2020-01-13

**Authors:** Wan-Jin Chang, Shuqiang Li

**Affiliations:** 1 Institute of Zoology, Chinese Academy of Sciences, Beijing 100101, China Institute of Zoology, Chinese Academy of Sciences Beijing China

**Keywords:** Borneo, Indonesia, Kalimantan, Philippines, Sulawesi, Vietnam

## Abstract

The genus *Psiloderces* Simon, 1892 is the second largest genus of Psilodercidae, a family restricted to Asia, occurring from East India to the Philippines. Fourteen new species of *Psiloderces* from Indonesia, the Philippines, and Vietnam are described: *P. heise***sp. nov.** (♂♀), *P. gawanaensis***sp. nov.** (♂♀), *P. bontocensis***sp. nov.** (♀), *P. cuyapoensis***sp. nov.** (♂♀), *P. xichang***sp. nov.** (♂♀), *P. cattienensis***sp. nov.** (♂♀), *P. pingguo***sp. nov.** (♂♀), *P. palopoensis***sp. nov.** (♂♀), *P. penajamensis***sp. nov.** (♂), *P. grohotensis***sp. nov.** (♂♀), *P. bangkiraiensis***sp. nov.** (♂♀), *P. bolang***sp. nov.** (♂♀), *P. wangou***sp. nov.** (♂♀), and *P. malinoensis***sp. nov.** (♂♀). Types are deposited in the Institute of Zoology, Chinese Academy of Sciences (IZCAS) in Beijing.

## Introduction

The spider family Psilodercidae Machado, 1951 was treated as a subfamily of Ochyroceratidae Fage, 1912 until Wunderlich (2004) elevated it to the family level. Currently, it is known by 11 genera and 151 species distributed in Southern Asia from India to the Philippines (Li and Quan 2017; WSC 2019). The number of species in Psilodercidae has increased nearly threefold during the 21^th^ century (Platnick 2000). More than half of psilodercid genera have been described only recently: *Flexicrurum* Tong & Li, 2007, *Luzonacera* Li & Li, 2017, *Priscaleclercera* Wunderlich, 2017, *Qiongocera* Li & Li, 2017, *Relictocera* Li & Li, 2017, *Sinoderces* Li & Li, 2017, and *Thaiderces* Li & Li, 2017.

At present, 24 species of *Psiloderces* Simon, 1892 are known from China, Indonesia, Malaysia, the Philippines, Sri Lanka, and Thailand (WSC 2019). About three quarters of the known species are found in Indonesia and Thailand. Up to now, the type species *Psiloderces egeria* Simon, 1892 and another four species, i.e., *P. elasticus* Brignoli, 1975, *P. kalimantan* Deeleman-Reinhold, 1995, *P. penaeorum* Deeleman-Reinhold, 1995, and *P. tesselatus* Deeleman-Reinhold, 1995 are known only from female specimens, and *P. dicellocerus* Li, Li & Jäger, 2014, *P. fredstonei* Deeleman-Reinhold, 1995, *P. incomptus* Wang & Li, 2013, *P. limosa* Deeleman-Reinhold, 1995, and *P. nasicornis* Baert, 1988 are known only from male specimens. Deeleman-Reinhold (1995) provisionally classified *Psiloderces* species into nine groups based on a combination of characteristics of the vulva and palp.

During the examination of a spider collection from Southeast Asia, we found fourteen new species of *Psiloderces* from Kalimantan and Sulawesi (Indonesia), the Philippines, and Vietnam. The goals of this paper are to provide detailed descriptions of the new species with images of their copulatory organs and chelicerae, as well as discuss their placement in the aforementioned species groups (Deeleman-Reinhold 1995).

## Materials and methods

Types are deposited in the Institute of Zoology, Chinese Academy of Sciences (IZCAS) in Beijing. All specimens were observed and preserved in 95% ethanol. The specimens were measured and examined using a Leica M205 C stereomicroscope, and further morphological details were observed with an Olympus BX41 compound microscope. The left palp of the male was detached for further examination (except for *Psiloderces penajamensis*, the right palp was detached). Carapace length was measured excluding the clypeus. The internal genitalia and male palpal bulb were dissected and immersed in lactic acid. An Olympus C7070 wide zoom digital camera (7.1 megapixels) mounted on an Olympus SZX12 stereomicroscope was used to take photos in different focal planes. The photos were then transferred to Helicon Focus 6.7.1 image stacking software to improve depth of field before further revision with Adobe Photoshop CC 2014. Leg measurements are shown as total length (femur, patella, tibia, metatarsus, and tarsus). Leg segments were measured from their retrolateral side. All measurements are given in millimetres (mm). All terminology follows Li et al. (2014).

## Taxonomy

### Family Psilodercidae Machado, 1951

#### Genus Psiloderces

Taxon classificationAnimaliaAraneaePsilodercidae

Simon, 1892

85098FAE-2B14-557F-B0EC-6A196FAFC13C

Psiloderces Simon, 1892: 40.
Psiloderces : Deeleman-Reinhold, 1995: 7.

##### Type species.

*Psiloderces egeria* Simon, 1892 from the Philippines.

##### Emended diagnosis.

*Psiloderces* resembles *Thaiderces* by having a shallow, dark brown fovea, a cheliceral promargin with a lamina bearing 3 triangular extensions, the retromargin with 2 small teeth, and the anterior part of the thoracic region is distinctly elevated, but it can be differentiated by the following characters: 1) the presence of a cymbial protrusion (vs. absent in *Thaiderces*); 2) the presence of an inconspicuous clypeal protrusion in *P. enigmatus* Deeleman-Reinhold, 1995, *P. pulcher* Deeleman-Reinhold, 1995, *P. incomptus*, *P. nasicornis*, *P. gawanaensis* sp. nov., *P. cuyapoensis* sp. nov., *P. xichang* sp. nov., *P. cattienensis* sp. nov., *P. pingguo* sp. nov., and *P. penajamensis* sp. nov. (vs. absent in *Thaiderces*); and 3) the presence or absence of a laminar apophysis or a bulge. If present, then the embolus and laminar apophysis are not separated basally, or the bulge is separated basally from the embolus (vs. absence of bulge and such combination of embolus and laminar apophysis in *Thaiderces*).

##### Remarks.

The type species of the genus is known from a female, and the female genitalia is insufficient for genus identification. However, the somatic morphology together with the morphology of male of the species and DNA barcoding data all confirm that these species belong to the genus *Psiloderces*.

##### Species groups.

Nine *Psiloderces* species groups were established by Deeleman-Reinhold (1995). They are:

*althepoides*-group: endogyne with a pair of sessile spermathecae; bulb pyriform, with tapering tip, extremely long legs. Species included: *P. althepoides* Deeleman-Reinhold, 1995 (♂♀).

*egeria*-group: endogyne with a pair of sausage-like spermathecae; bulb syringiform. Species included: *P. palopoensis* sp. nov. (♂♀) and *P. egeria* (♀) (type species).

*enigmatus*-group: endogyne with a pair of sessile or sausage-like spermathecae; embolus arises distally from bulb; male with clypeus projection. Species included: *P. gawanaensis* sp. nov. (♂♀), *P. cuyapoensis* sp. nov. (♂♀), *P. xichang* sp. nov. (♂♀), *P. penajamensis* sp. nov. (♂), *P. enigmatus* (♂♀), *P. pulcher* (♂♀), and *P. tesselatus* (♀).

*howarthi*-group: endogyne with membranous projection, guiding ridges, and a pair of sausage-like, sessile spermathecae; bulb syringiform. Species included: *P. howarthi* Deeleman-Reinhold, 1995 (♂♀).

*leucopygius*-group: endogyne with 2 pairs of pedunculated spermathecae; palp with simple syringiform bulb. Species included: *P. pingguo* sp. nov. (♂♀), *P. elasticus* (♀), *P. dicellocerus* (♂), *P. incomptus* (♂), *P. leucopygius* Deeleman-Reinhold, 1995 (♂♀), *P. vallicola* Deeleman-Reinhold, 1995 (♂♀), *P. limosa* (♂), *P. coronatus* Deeleman-Reinhold, 1995 (♂♀), and *P. penaeorum* (♀).

*ligula*-group: endogyne with sausage-like spermathecae; male with coiled embolus with spatula-shaped tip. Species included: *P. ligula* Baert, 1988 (♂♀).

*longipalpis*-group: endogyne with a pair of sessile spermathecae; bulb constricted medially, embolus arises distally; male without clypeus protrusion. Species included: *P. grohotensis* sp. nov. (♂♀), *P. bangkiraiensis* sp. nov. (♂♀), *P. bolang* sp. nov. (♂♀), *P. wangou* sp. nov. (♂♀), *P. malinoensis* sp. nov. (♂♀), *P. longipalpis* Baert 1988 (♂♀), *P. nasicornis* (♂), *P. torajanus* Deeleman-Reinhold, 1995 (♂♀), *P. leclerci* Deeleman-Reinhold, 1995 (♂♀), and *P. kalimantan* (♀).

*mulcatus*-group: endogyne with a pair of stalked or pedunculated spermathecae; bulb with indentation medially, embolus arises distally. Species included: *P. heise* sp. nov. (♂♀) and *P. bontocensis* sp. nov. (♀).

*septentrionalis*-group: endogyne with 2 pairs of sessile sausage-like spermathecae; bulb simple syringiform. Species included: *P. cattienensis* sp. nov. (♂♀), *P. septentrionalis* Deeleman-Reinhold, 1995 (♂♀), *P. suthepensis* Deeleman-Reinhold, 1995 (♂♀), *P. albostictus* Deeleman-Reinhold, 1995 (♂♀), and *P. fredstonei* (♂).

##### Distribution.

The genus is known from China, Vietnam to the Philippines, and south to Indonesia (Kalimantan and Sulawesi).

#### Psiloderces heise

Taxon classificationAnimaliaAraneaePsilodercidae

Li & Chang sp. nov.

E282EA5F-36D2-511C-A93F-E2AC23D88020

http://zoobank.org/301F1310-5C27-488B-A335-2C26D5CFCC9B

**Figure 1.**
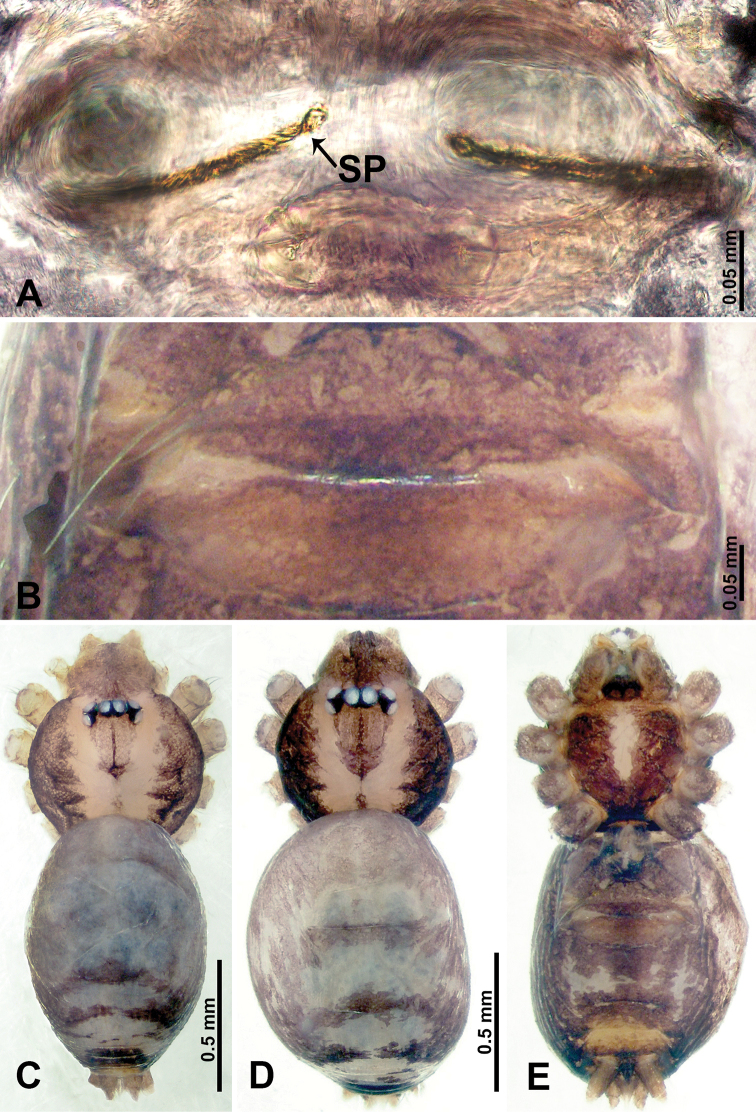
*Psiloderces heise* sp. nov., male holotype and female paratype. **A** Endogyne, dorsal view **B** female epigastric area, ventral view **C** male habitus, dorsal view **D** female habitus, dorsal view **E** female habitus, ventral view. Abbreviation: SP = spermatheca.

**Figure 2.**
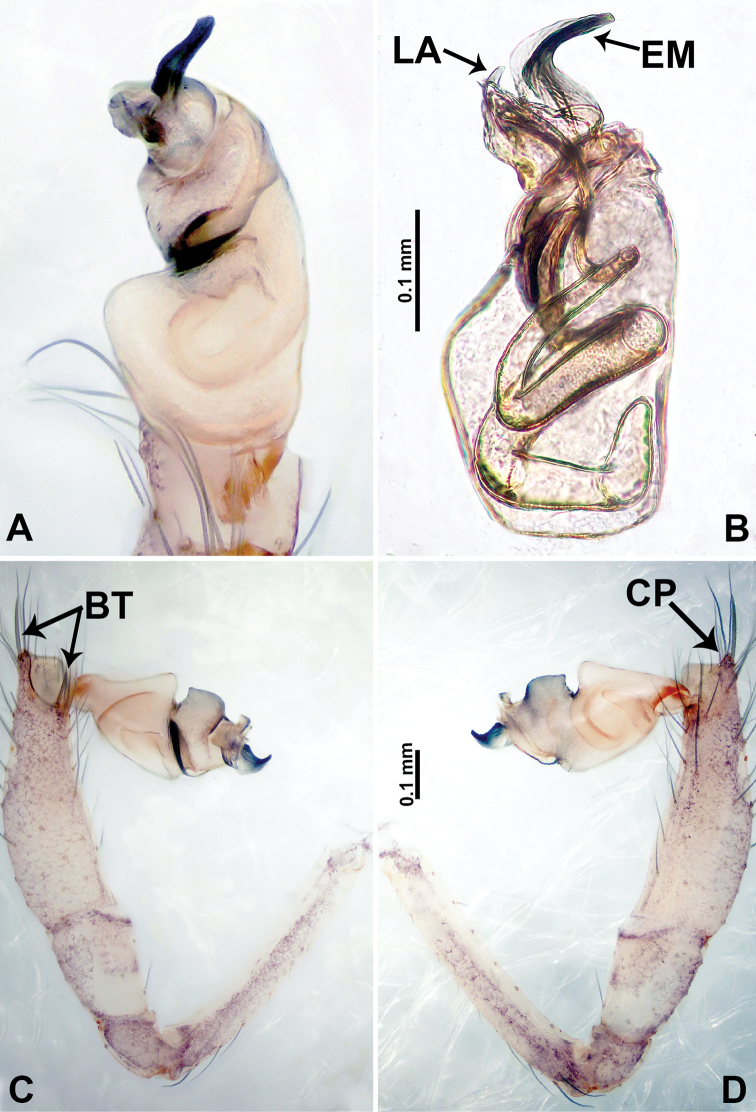
*Psiloderces heise* sp. nov., male holotype. **A** Palp, ventral view **B** bulb, ventral view **C** palp, prolateral view **D** palp, retrolateral view. Abbreviations: BT = bristle, CP = cymbial protrusion, EM = embolus, LA = laminar apophysis.

Figs 1 , 2 , 28C , 30

##### Types.

***Holotype:*** ♂ (IZCAS), Philippines, Luzon Island, Tarlac Province, Tarlac City, near Monasterio de Tarlac, 15°26.8998'N, 120°25.6710'E, 123 m, 20.V.2015, F. Ballarin & Y. Li. ***Paratype***: 1♀ (IZCAS), same data as holotype.

##### Etymology.

The species name is a noun in apposition derived from the Chinese pinyin “hēisè” (black) and refers to the unique black color of the embolus.

##### Diagnosis.

Males of *P. heise* sp. nov. can be distinguished from all other species of the genus by the structure of the bulb with a distinct prolateral indentation (Fig. 2C, D), a cymbial protrusion with a right-angled attachment (Fig. 2C), and the thick and dark embolus (vs. the absence of an indentation, a right-angled attachment, and different colored embolus in congeners); females can be differentiated from congeners by a pair of horizontally stalked spermathecae that width almost equally wide with globose distal part (Fig. 1A).

##### Description.

**Male** (Holotype). Total length 1.46; carapace 0.56 long, 0.63 wide; abdomen 0.90 long, 0.70 wide. Carapace round and brown, with 3 longitudinal dark brown bands, median band half length of carapace, lateral bands eight times wider than median band (Fig. 1C). Chelicerae pale brown (Fig. 28C). Clypeus slanting, brown. Endites brown. Labium dark brown. Sternum dark brown, delimiting light brown band medially. Abdomen elongated, dorsum with purplish stripes posteriorly, antero-ventrally brown with pair of circular patches followed by semi-circular patch, posterior half with indistinct brown and light brown patterns. Legs uniformly brown; measurements: I 3.86 (1.00, 0.20, 1.09, 1.02, 0.55), II 4.38 (1.25, 0.20, 1.20, 1.10, 0.63), III 3.75 (1.00, 0.25, 1.00, 1.00, 0.50), IV 5.31 (1.41, 0.20, 1.60, 1.40, 0.70). Palp (Fig. 2A–D): femur slender, 3 times longer than patella; patella not swollen; tibia 2 times shorter than femur but almost as wide as cymbium; cymbium pale, 2 times wider than femur but almost as long as femur, cymbial protrusion with right-angled stick-out attachment and distinct bristles basally and anteriorly; bulb light yellow, pyriform, with slight indentation promarginally, laminar apophysis and embolus arise distally; laminar apophysis not separated from embolus basally, shorter than embolus; embolus thicker and darker than laminar apophysis, bent apically (Fig. 2B).

**Female** (Paratype). General features and coloration similar to those of male (Fig. 1D, E). Measurements: total length 1.40; carapace 0.50 long, 0.47 wide; abdomen 0.90 long, 0.63 wide. Leg measurements: I 4.16 (1.10, 0.16, 1.25, 1.02, 0.63), II 3.45 (0.90, 0.20, 0.94, 0.86, 0.55), III 3.72 (1.00, 0.13, 1.10, 1.02, 0.47), IV 2.81 (0.70, 0.16, 0.78, 0.70, 0.47). Endogyne (Fig. 1A): transverse stalked spermathecae bearing globose distal parts, heads almost as wide as stalks, stalks 10 times longer than heads.

##### Distribution.

Known only from the type locality (Fig. 30).

#### Psiloderces gawanaensis

Taxon classificationAnimaliaAraneaePsilodercidae

Li & Chang sp. nov.

5B15B336-501E-5758-861C-7B093B37C528

http://zoobank.org/15281939-437A-4A72-B5E3-ABF4BA47B4A7

**Figure 3.**
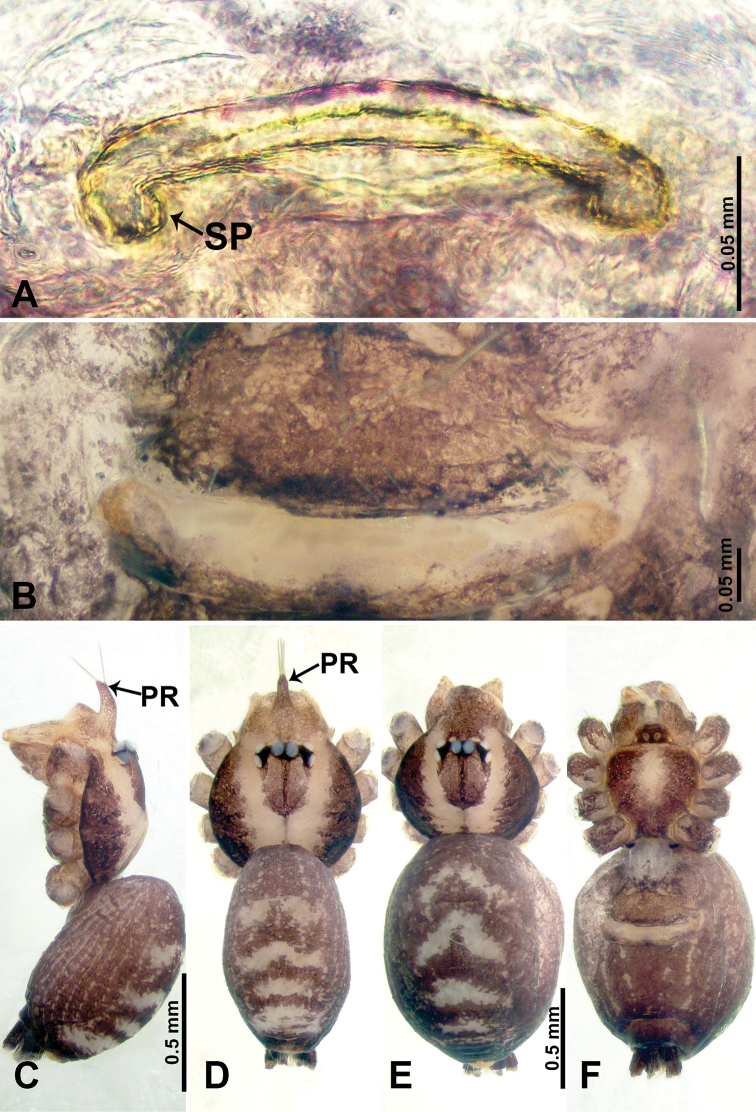
*Psiloderces gawanaensis* sp. nov., male holotype and female paratype. **A** Endogyne, dorsal view **B** female epigastric area, ventral view **C** male habitus, lateral view **D** male habitus, dorsal view **E** female habitus, dorsal view **F** female habitus, ventral view. Abbreviations: PR = clypeal protrusion, SP = spermatheca.

**Figure 4.**
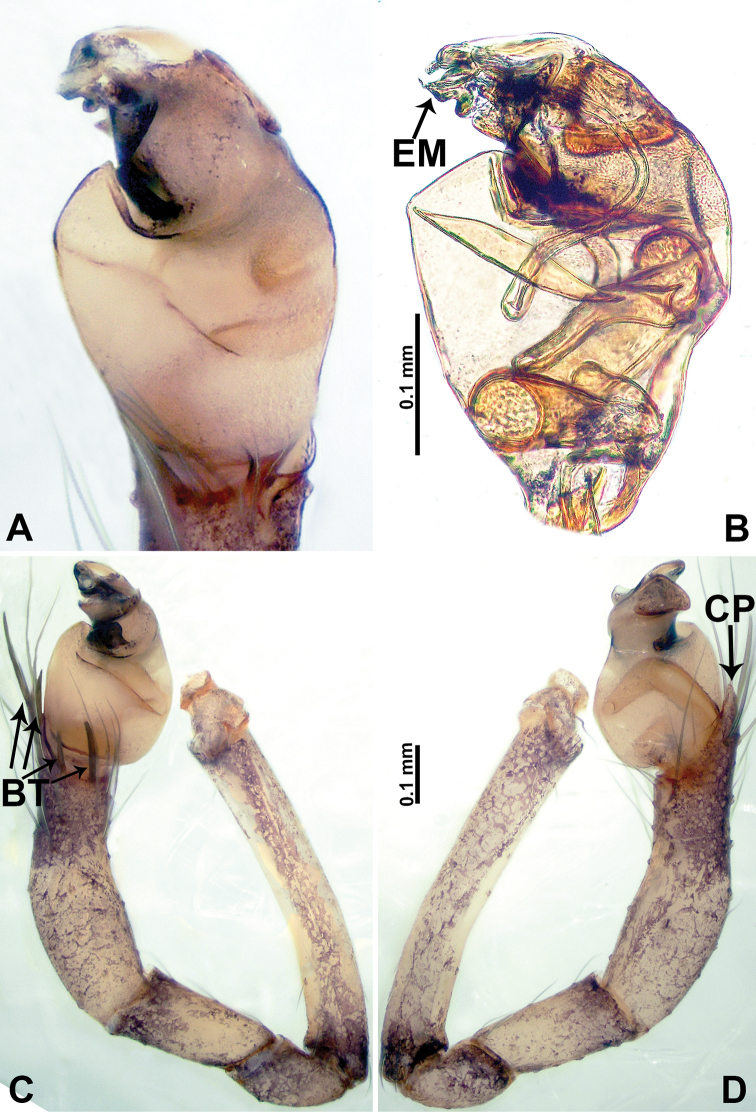
*Psiloderces gawanaensis* sp. nov., male holotype. **A** Palp, ventral view **B** bulb, ventral view **C** palp, prolateral view **D** palp, retrolateral view. Abbreviations: BT = bristle, CP = cymbial protrusion, EM = embolus.

Figs 3 , 4 , 28F , 30

##### Types.

***Holotype:*** ♂ (IZCAS), Philippines, Luzon Island, Mountain Province, Bontoc Town, road to Gawana Town, 17°3.6396'N, 121°3.0402'E, 1674 m, 26.V.2015, F. Ballarin & Y. Li. ***Paratype***: 1♀ (IZCAS), same data as holotype.

##### Etymology.

The species name is an adjective referring to the type locality.

##### Diagnosis.

Males of *P. gawanaensis* sp. nov. can be distinguished from congeners by the human heart-shaped bulb (inverted pyriform) (Fig. 4B) bearing a threadlike embolus, the presence of 4 distinct bristles on the cymbial protrusion, and the clypeal projection bearing a few setae (Fig. 4C, D) (vs. absence or less than 4 bristles on cymbial protrusion); females can be differentiated from congeners by the widely spaced, horseshoe-shaped spermathecae (Fig. 3A).

##### Description.

**Male** (Holotype). Total length 1.54; carapace 0.60 long, 0.63 wide; abdomen 0.94 long, 0.56 wide. Carapace wider than long, brown, with 3 longitudinal dark brown bands, median band half length of carapace, median band two times wider than lateral band (Fig. 3D). Chelicerae pale brown (Fig. 28F). Clypeus dark brown, with long and slightly bent medial projection, bearing few setae apically (Fig. 3C, D). Endites dark brown. Labium dark brown delimiting pair of light brown circular spots. Sternum dark brown, delimiting light brown band medially. Abdomen elongated, dorsum with brown stripes, antero-ventrally brown with elliptical patch, posterior part with indistinct brown pattern. Legs uniformly brown; measurements: I 5.21 (1.33, 0.25, 1.63, 1.20, 0.80), II 4.03 (1.09, 0.20, 1.25, 0.94, 0.55), III 3.49 (0.94, 0.20, 0.94, 0.86, 0.55), IV 4.88 (1.25, 0.25, 1.38, 1.25, 0.75). Palp (Fig. 4A–D): femur slender, 4 times longer than patella; patella not swollen; tibia 3 times shorter than femur; cymbium purplish distally, 2 times shorter than wide, and 2 times wider than femur, protrusion with 4 distinct bristles basally and anteriorly; bulb light brown, inverted pyriform with threadlike embolus arising distally (Fig. 4B).

**Female** (Paratype). General features and coloration similar to those of male (Fig. 3E, F). Measurements: total length 1.64; carapace 0.70 long, 0.60 wide; abdomen 0.94 long, 1.20 wide. Leg measurements: I 5.23 (1.25, 0.25, 1.60, 1.25, 0.88), II 4.30 (1.10, 0.20, 1.30, 1.00, 0.70), III missing, IV 5.24 (1.30, 0.23, 1.60, 1.33, 0.78). Endogyne (Fig. 3A): encircled spermathecae resemble a horseshoe, curving downwards (Fig. 3A).

##### Distribution.

Known only from the type locality (Fig. 30).

#### Psiloderces bontocensis

Taxon classificationAnimaliaAraneaePsilodercidae

Li & Chang sp. nov.

D3417E06-D3AF-51C1-A929-46E824590E82

http://zoobank.org/F99F7890-8D40-449E-900D-E3B3FD7FB52E

Figs 5 , 28E , 30

##### Types.

***Holotype:*** ♀ (IZCAS), Philippines, Luzon Island, Mountain Province, Bontoc Town, road to Banawe Town, 16°59.6630'N, 121°1.1120'E, 1470 m, 25.V.2015, F. Ballarin & Y. Li.

##### Etymology.

The species name is an adjective referring to the type locality.

##### Diagnosis.

**Figure 5.**
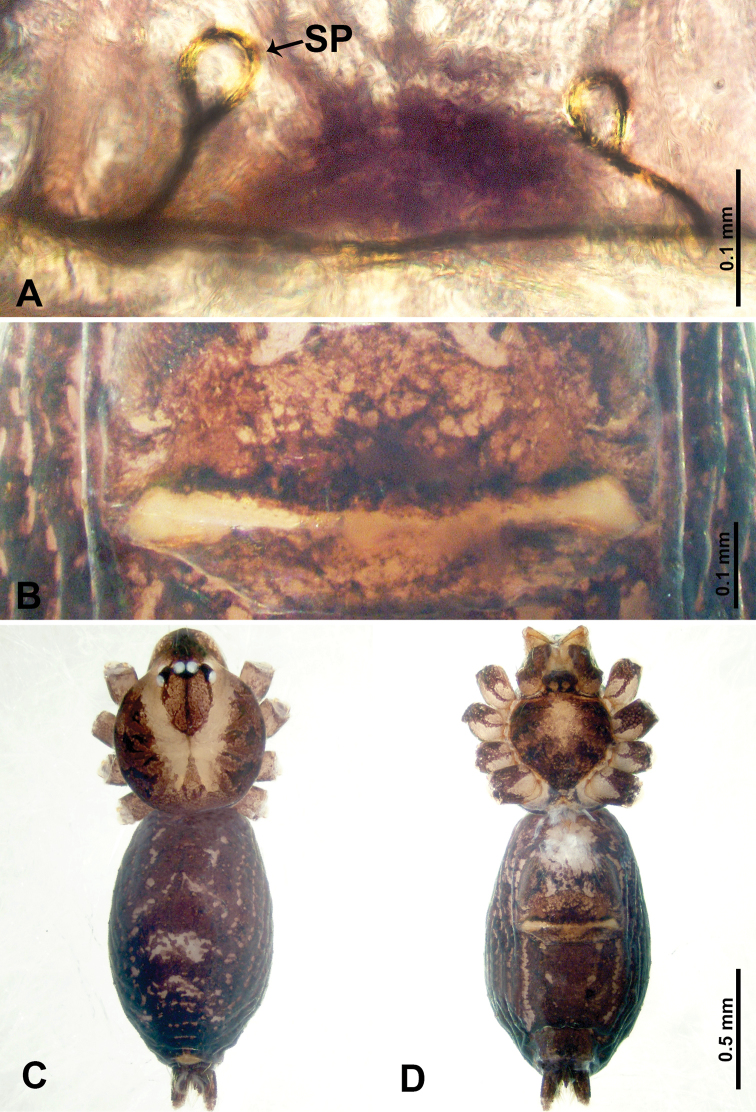
*Psiloderces bontocensis* sp. nov., female paratype. **A** Endogyne, dorsal view **B** female epigastric area, ventral view **C** female habitus, dorsal view **D** female habitus, ventral view. Abbreviation: SP = spermatheca.

The female of *P. bontocensis* sp. nov. can be distinguished from others by a pair of stalked spermathecae with bulbous distal parts, spermathcae almost 8 times wider than stalk, resembling balloons (vs. balloon-shaped spermathecae absent in congeners) (Fig. 5A).

##### Description.

**Female** (Holotype). Total length 2.00; carapace 0.60 long, 0.70 wide; abdomen 1.40 long, 0.81 wide. Carapace almost round, wider than long, brown, with 3 longitudinal dark brown bands, median band extends only half length of carapace, lateral bands almost as wide as the middle band (Fig. 5C). Chelicerae brown (Fig. 28E). Clypeus slanting, dark brown. Endites dark brown, light brown basally. Labium dark brown, delimiting pair of light brown circular spots. Sternum dark brown, delimiting light brown patch medially. Abdomen elongated, dorsum with mixture of dark and pale brown forming indistinct pattern (Fig. 5C), antero-ventrally dark brown with semi-circular brown patch medially, posterior part with light brown dots laterally (Fig. 5D). Legs uniformly brown; measurements: I 5.20 (1.25, 0.25, 1.60, 1.30, 0.80) II 4.32(1.09, 0.20, 1.25, 1.09, 0.69), III 3.48 (0.88, 0.20, 0.90, 0.90, 0.60), IV 5.15(1.25, 0.20, 1.60, 1.30, 0.80). Endogyne (Fig. 5A): stalked spermathecae with bulbous distal part, stalks two times longer than bulbous part, bulbous part 8 times wider than stalks (Fig. 5A).

**Male**. Unknown.

##### Distribution.

Known only from the type locality (Fig. 30).

#### Psiloderces cuyapoensis

Taxon classificationAnimaliaAraneaePsilodercidae

Li & Chang sp. nov.

42FCA5DD-AF09-5F5C-AE20-4633E6CFD55B

http://zoobank.org/7E6FFA31-1180-4D0D-9F08-AF683147B829

**Figure 6.**
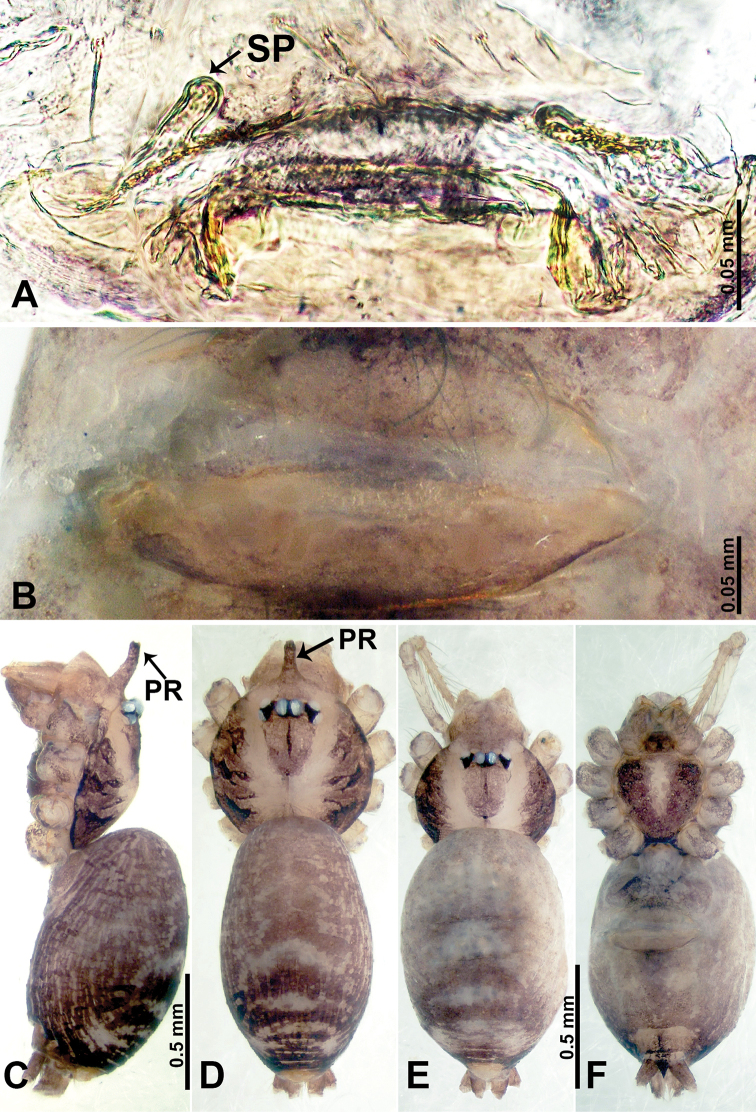
*Psiloderces cuyapoensis* sp. nov., male holotype and female paratype. **A** Endogyne, dorsal view **B** female epigastric area, ventral view **C** male habitus, lateral view **D** male habitus, dorsal view **E** female habitus, dorsal view **F** female habitus, ventral view. Abbreviations: PR = clypeal protrusion, SP = spermatheca.

**Figure 7.**
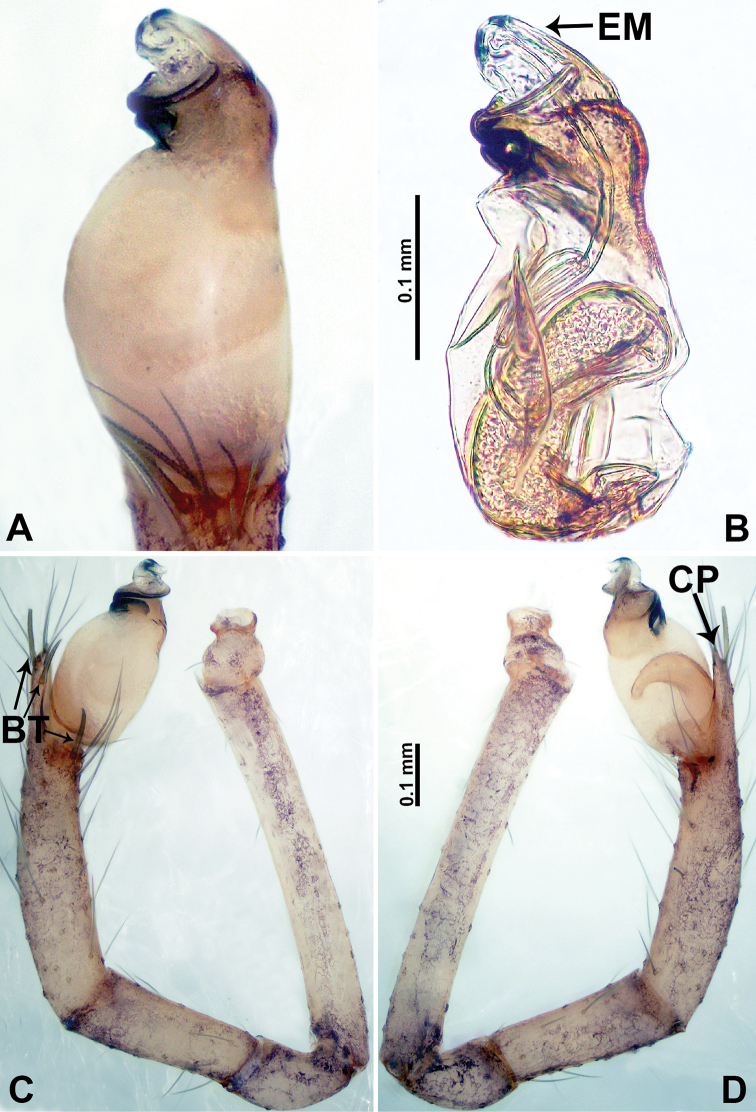
*Psiloderces cuyapoensis* sp. nov., male holotype. **A** Palp, ventral view **B** bulb, ventral view **C** palp, prolateral view **D** palp, retrolateral view. Abbreviations: BT = bristle, CP = cymbial protrusion, EM = embolus.

Figs 6 , 7 , 28D , 30

##### Types.

***Holotype:*** ♂ (IZCAS), Philippines, Luzon Island, Nueva Ecija Province, Cuyapo City, 15°47.8086'N, 120°39.3294'E, 60 m, 22.V.2015, F. Ballarin & Y. Li. ***Paratype***: 1♀ (IZCAS), same data as holotype.

##### Etymology.

The species name is an adjective referring to the type locality.

##### Diagnosis.

Males of *P. cuyapoensis* sp. nov. resemble those of *P. xichang* sp. nov. but can be distinguished by the rounded embolus (vs. wavy embolus), 3 distinct bristles on the cymbial protrusion (vs. 2 distinct bristles on cymbial protrusion), length of palp ca 6 times the length of bulb (vs. extremely slender and long palp, ca 10 times the length of bulb (Fig. 9C, D), and coloration of male and female are pale brown (vs. coloration of male and female are darker brown); females can be distinguished by tubular spermathecae (vs. earlobe-shaped spermathecae).

##### Description.

**Male** (Holotype). Total length 1.60; carapace 0.60 long, 0.67 wide; abdomen 1.00 long, 0.78 wide. Carapace round and brown, with 3 longitudinal dark brown bands, median band half carapace length, median band almost as wide as the lateral bands (Fig. 6D). Chelicerae pale brown (Fig. 28D). Clypeus dark brown, with long, slightly bent medial projection (Fig. 6C, D). Endites light brown. Labium dark brown delimiting pair of light brown circular dots. Sternum dark brown, delimiting light brown band medially. Abdomen elongated, dorsum with brown stripes, antero-ventrally pale brown with elliptical patch, posterior with purplish patterns. Legs uniformly brown; measurements: I–III missing, IV 5.21 (1.33, 0.25, 1.50, 1.38, 0.75). Palp (Fig. 7A–D): femur slender, 4 times longer than patella; patella not swollen; tibia 1.50 times shorter than femur; cymbium 2 times shorter and almost as wide as femur, protrusion with 3 distinct bristles basally and anteriorly; bulb pale brown, bulging pyriform with embolus arising distally; embolus rounded and blunt (Fig. 7B).

**Female** (Paratype). General features and coloration similar to those of male (Fig. 6E, F). Measurements: total length 1.64; carapace 0.47 long, 0.63 wide; abdomen 1.17 long, 0.78 wide. Leg measurements: I–IV missing. Endogyne (Fig. 6A): tubular spermathecae with indistinct concave ducts posteriorly (Fig. 6A).

##### Distribution.

Known only from the type locality (Fig. 30).

#### Psiloderces xichang

Taxon classificationAnimaliaAraneaePsilodercidae

Li & Chang sp. nov.

4F09801E-689F-53F9-8934-64CFB0EC23E2

http://zoobank.org/A219F896-1005-440F-B783-6E8A08B3252E

**Figure 8.**
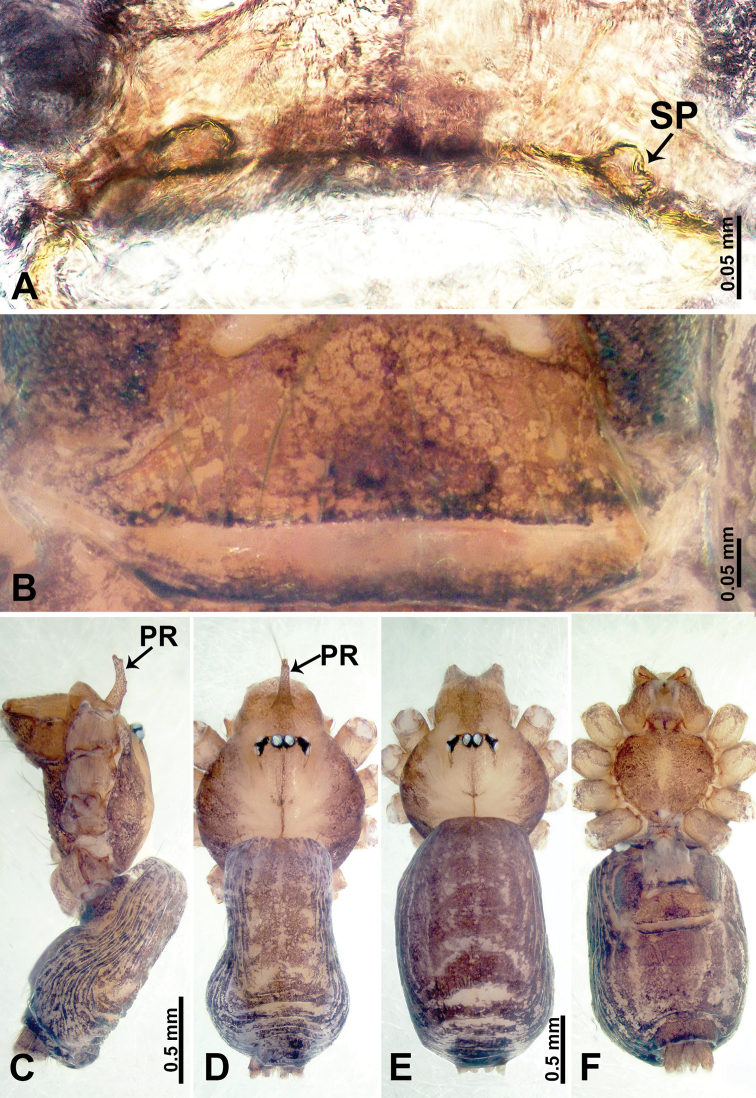
*Psiloderces xichang* sp. nov., male holotype and female paratype. **A** Endogyne, dorsal view **B** female epigastric area, ventral view **C** male habitus, lateral view **D** male habitus, dorsal view **E** female habitus, dorsal view **F** female habitus, ventral view. Abbreviations: PR = clypeal protrusion, SP = spermatheca.

**Figure 9.**
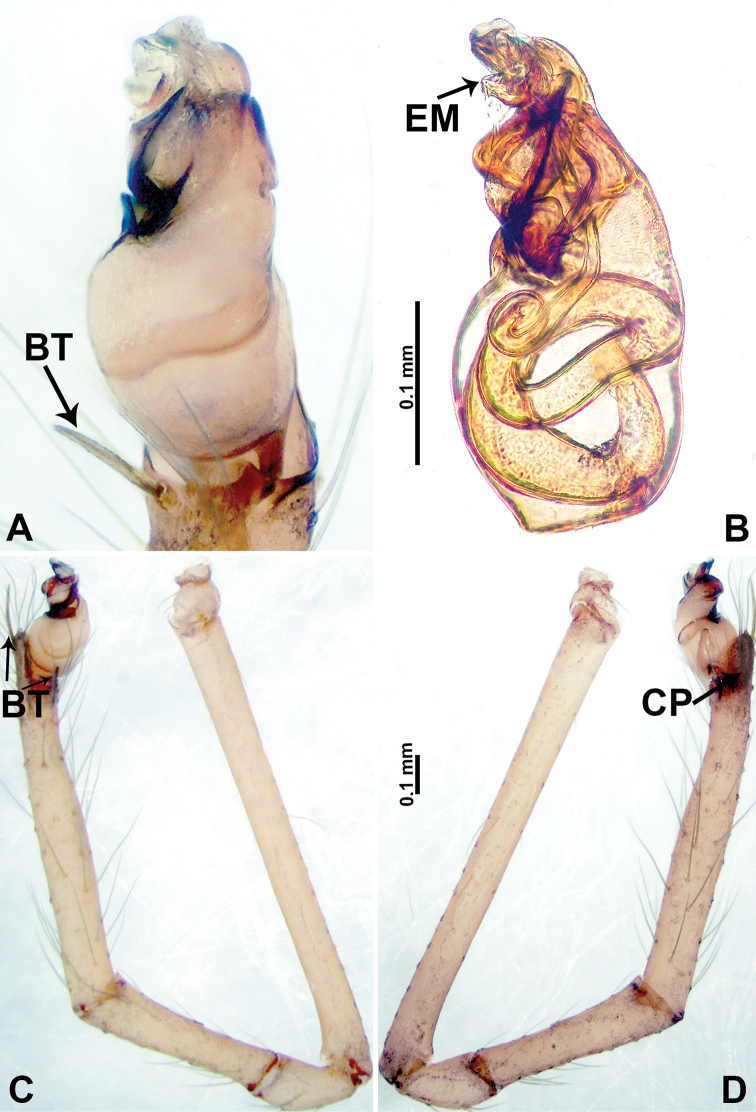
*Psiloderces xichang* sp. nov., male holotype. **A** Palp, ventral view **B** bulb, ventral view **C** palp, prolateral view **D** palp, retrolateral view. Abbreviations: BT = bristle, CP = cymbial protrusion, EM = embolus.

Figs 8 , 9 , 28G , 30

##### Types.

***Holotype:*** ♂ (IZCAS), Philippines, Luzon Island, Rizal Province, Quezon City, Rodriguez area, Montalban Village, Pamitinan Cave, 14°43.7916'N, 121°11.3580'E, 102 m, 5.VI.2015, F. Ballarin & Y. Li. ***Paratype***: 1♀ (IZCAS), same data as holotype.

##### Etymology.

The species name is a noun in apposition derived from the Chinese pinyin “xìcháng” (slender and long) and refers to the extremely slender palp structures.

##### Diagnosis.

Diagnostic features of males and females are discussed in the diagnosis of *P. cuyapoensis* sp. nov.

##### Description.

**Male** (Holotype). Total length 2.00; carapace 0.70 long, 0.90 wide; abdomen 1.30 long, 1.00 wide. Carapace round and brown, with 2 longitudinal dark brown bands laterally, medially with dark brown line (Fig. 8D). Chelicerae brown (Fig. 28G). Clypeus dark brown, with long, slightly bent medial projection, bearing few setae (Fig. 8C, D). Endites dark brown, light brown basally. Labium dark brown delimiting pair of light brown circular dots. Sternum dark brown, delimiting light brown band medially. Abdomen elongated, dorsum with complex brown patterns, antero-ventrally dark brown with pair of circular patches followed by semi-circular patch, posterior with indistinct dark brown and light brown patterns. Legs uniformly brown; measurements: I 10.30 (2.80, 0.25, 3.20, 2.80, 1.25), II 8.91 (2.40, 0.31, 2.60, 2.60, 1.00), III 6.23(1.72, 0.25, 1.75, 1.63, 0.88), IV 9.34 (2.25, 1.00, 2.66, 2.34, 1.09). Palp equally wide throughout its length (Fig. 9A–D): femur extremely slender, 6 times longer than patella; patella not swollen; tibia 2.50 times shorter than femur; cymbium 2 times shorter than femur, protrusion with 2 distinct bristles basally and anteriorly; bulb pale brown, bulging pyriform with embolus arising distally; embolus irregular, resembles a petal (Fig. 9A).

**Female** (Paratype). General features and coloration similar to those of male (Fig. 8E, F). Measurements: total length 2.16; carapace 0.75 long, 0.86 wide; abdomen 1.41 long, 1.02 wide. Leg measurements: I 9.66 (2.60, 0.31, 3.00, 2.66, 1.09), II 7.91 (2.13, 0.31, 2.34, 2.19, 0.94), III 6.63 (1.63, 0.25, 1.50, 1.50, 1.75), IV 10.96 (2.80, 0.31, 3.60, 3.00, 1.25) Endogyne (Fig. 8A): widely spaced, earlobe-like spermathecae, ratio of spermathecae interdistances and spermathecae width 1:4 (Fig. 8A).

##### Distribution.

Known only from the type locality (Fig. 30).

#### Psiloderces cattienensis

Taxon classificationAnimaliaAraneaePsilodercidae

Li & Chang sp. nov.

44D14254-5A3D-5B7C-B2B3-F1F11D7FC185

http://zoobank.org/EA2D9AD0-68C6-406A-89AF-E33968EC8712

**Figure 10.**
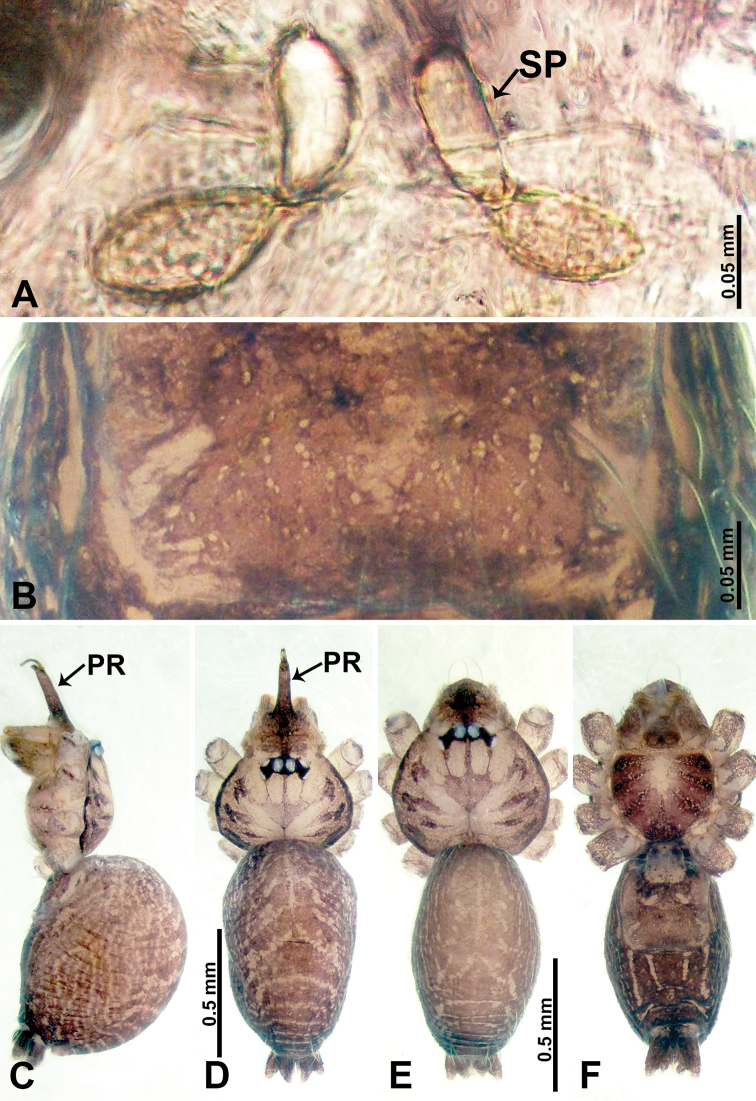
*Psiloderces cattienensis* sp. nov., male holotype and female paratype. **A** Endogyne, dorsal view **B** female epigastric area, ventral view **C** male habitus, lateral view **D** male habitus, dorsal view **E** female habitus, dorsal view **F** female habitus, ventral view. Abbreviations: PR = clypeal protrusion, SP = spermatheca.

**Figure 11.**
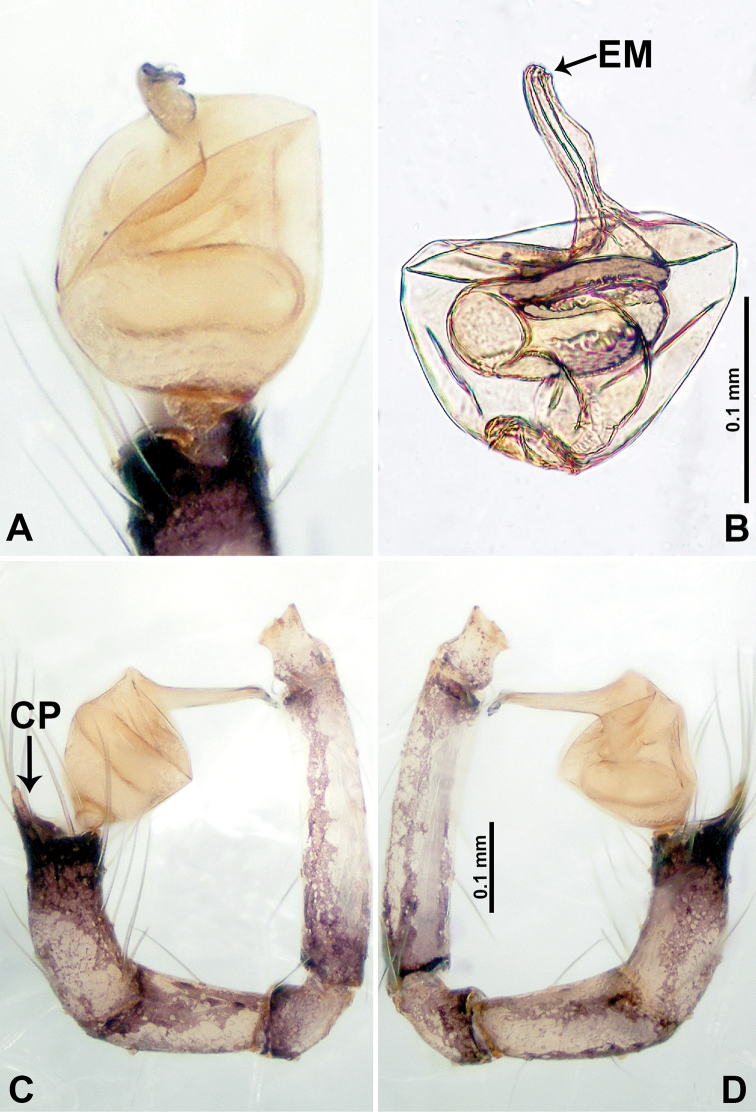
*Psiloderces cattienensis* sp. nov., male holotype. **A** Palp, ventral view **B** bulb, ventral view **C** palp, prolateral view **D** palp, retrolateral view. Abbreviations: CP = cymbial protrusion, EM = embolus.

Figs 10 , 11 , 28H , 30

##### Types.

***Holotype:*** ♂ (IZCAS), Vietnam, Dong Nai Province, Cat Tien National Park, 11°27.3620'N, 107°26.4980'E, 168 m, 4.IX.2015, Q. Zhao, Y. Li & Z. Chen. ***Paratype***: 1♀ (IZCAS), same data as holotype.

##### Etymology.

The species name is an adjective referring to the type locality.

##### Diagnosis.

Males of *P. cattienensis* sp. nov. resemble those of *P. pingguo* sp. nov. but can be distinguished by the relatively long embolus, which is equal to the length of the tegulum (vs. embolus two times shorter than the tegulum), the bulb is rather angular (vs. bulging); females can be distinguished by the merged tubular spermathecae (vs. stalked spermathecae globose distally).

##### Description.

**Male** (Holotype). Total length 1.32; carapace 0.44 long, 0.47 wide; abdomen 0.88 long, 0.50 wide. Carapace round and brown, with trident brown stripes medially and dark brown patches laterally (Fig. 10D). Chelicerae brown, cheliceral promargin with lamina bearing 2 triangular extensions (Fig. 10C, D). Endites dark brown. Labium dark brown delimiting pair of indistinct light brown circular dots. Sternum dark brown, delimiting light brown band medially. Abdomen elongated, dorsum with complex dark brown patterns, antero-ventrally dark brown with elliptical patch, posterior with 4 pairs of light brown vertical lines laterally and medially. Legs uniformly brown; measurements: I missing, II 3.45 (0.94, 0.13, 0.88, 0.94, 0.56), III 2.94 (0.81, 0.13, 0.81, 0.75, 0.44), IV missing. Palp (Fig. 11A–D): femur slender, 4 times longer than patella; patella not swollen; tibia 2 times shorter than femur; cymbium almost as long and wide as tibia, cymbial protrusion darkens distally; bulb pale brown, widely turbinate with embolus arising distally; embolus slightly bent at tip, almost as long as entire bulb (Fig. 11B).

**Female** (Paratype). General features and coloration similar to those of male (Fig. 10E, F). Measurements: total length 1.28; carapace 0.47 long, 0.55 wide; abdomen 0.81 long, 0.47 wide. Leg measurements: I 4.49 (1.13, 0.16, 1.41, 1.09, 0.70), II 3.53 (0.90, 0.20, 1.02, 0.86, 0.55), III 2.94 (0.75, 0.16, 0.81, 0.75, 0.47), IV 4.11 (1.00, 0.13, 1.33, 1.02, 0.63). Endogyne (Fig. 10A): spermathecae with lobes, cylindrical anteriorly and oval posteriorly, anterior lobes extended anteriorly and posterior lobes extended laterally.

##### Distribution.

Known only from the type locality (Fig. 30).

#### Psiloderces pingguo

Taxon classificationAnimaliaAraneaePsilodercidae

Li & Chang sp. nov.

1BBD34B7-BF33-5F9A-BB14-D15B43769153

http://zoobank.org/4D0C497A-B952-41BF-861B-A92F7113EE7F

**Figure 12.**
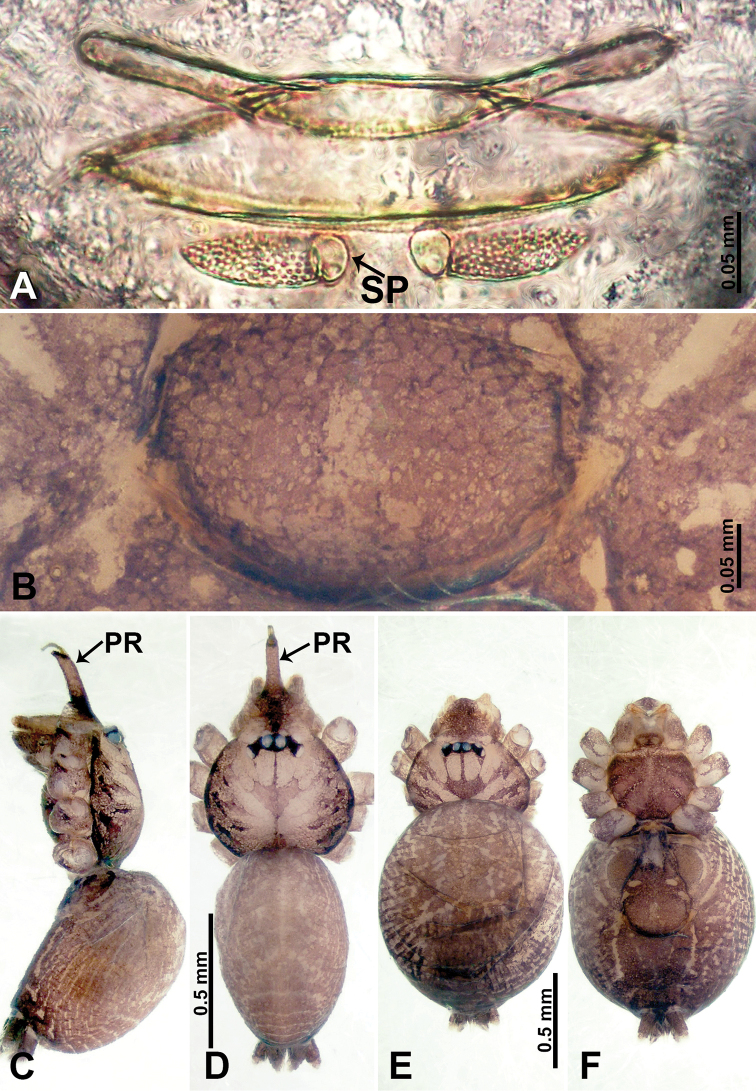
*Psiloderces pingguo* sp. nov., male holotype and female paratype. **A** Endogyne, dorsal view **B** female epigastric area, ventral view **C** male habitus, lateral view **D** male habitus, dorsal view **E** female habitus, dorsal view **F** female habitus, ventral view. Abbreviations: PR = clypeal protrusion, SP = spermatheca.

**Figure 13.**
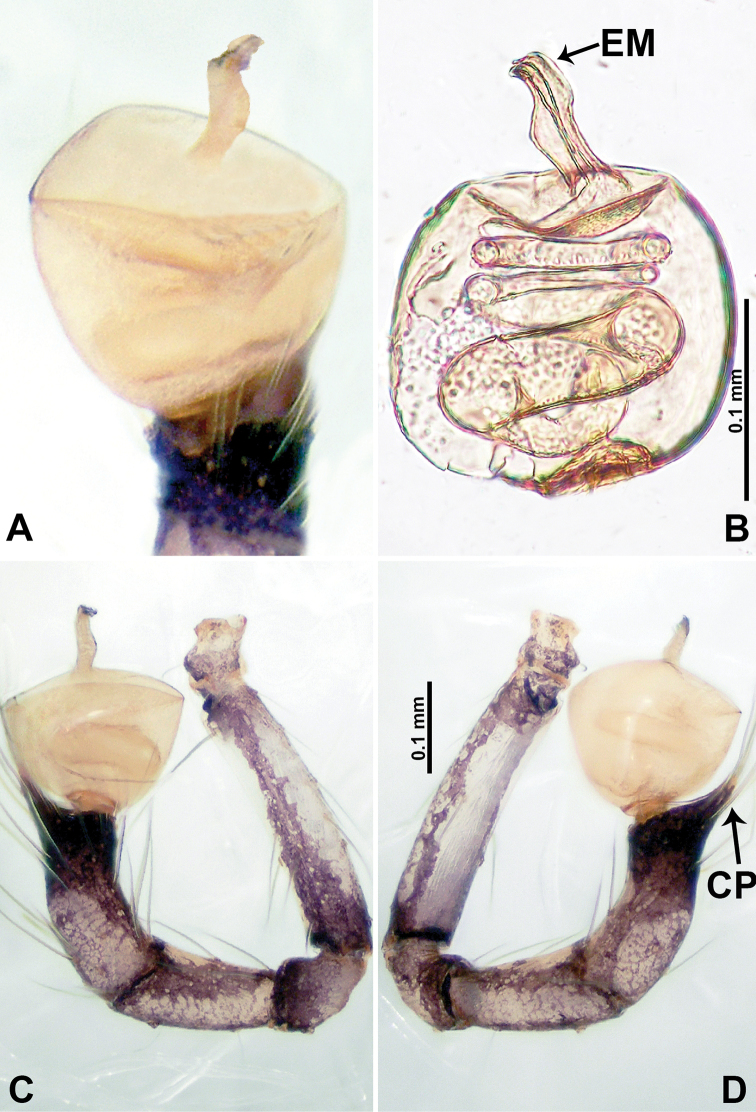
*Psiloderces pingguo* sp. nov., male holotype. **A** Palp, ventral view **B** bulb, ventral view **C** palp, prolateral view **D** palp, retrolateral view. Abbreviations: CP = cymbial protrusion, EM = embolus.

Figs 12 , 13 , 28I , 30

##### Types.

***Holotype:*** ♂ (IZCAS), Vietnam, Ninh Thuan Province, Nui Chua National Park, 11°43.9830'N, 107°11.1300'E, 102 m, 31.VIII.2015, Q. Zhao, Y. Li & Z. Chen ***Paratype***: 1♀ (IZCAS), same data as holotype.

##### Etymology.

The species name is a noun in apposition derived from the Chinese pinyin “píngguǒ” (apple) and refers to the structure of the bulb which resembles an apple.

##### Diagnosis.

Diagnostic features of the males and females are discussed in the diagnosis of *P. cattienensis* sp. nov.

##### Description.

**Male** (Holotype). Total length 1.25; carapace 0.47 long, 0.47 wide; abdomen 0.78 long, 0.47 wide. Carapace round and brown, with trident brown stripes medially and dark brown patches laterally (Fig. 12D). Chelicerae brown, cheliceral promargin with lamina bearing 2 triangular extensions (Fig. 28I). Clypeus dark brown, with bifurcate medial projection (Fig. 12C, D). Endites brown, light brown basally. Labium dark brown, delimiting pair of light brown circular dots. Sternum dark brown. Abdomen elongated, dorsum with complex brown patterns, antero-ventrally dark brown with pair of kidney-shaped patches followed by ovate patch, posterior with light brown vertical lines laterally. Legs uniformly brown; measurements: I–IV missing. Palp (Fig. 13A–D): femur slender, 3 times longer than patella; patella not swollen; tibia 1.5 times shorter than femur; cymbium almost as long and wide as tibia, protrusion darkens distally; bulb pale yellow, wide and cuneate with embolus arising medially; embolus slightly bent, half length of tegulum (Fig. 13B).

**Female** (Paratype). General features and coloration similar to those of male (Fig. 12E, F). Measurements: total length 1.56; carapace 0.47 long, 0.55 wide; abdomen 1.09 long, 0.86 wide. Leg measurements: I–IV missing. Endogyne (Fig. 12A): spermathecae stalked, with globose distal part, globose part almost as wide as stalk, stalks 4 times longer than globose part, spermathecae anteriorly elliptical with a pair of tubular ducts (Fig. 12A).

##### Distribution.

Known only from the type locality (Fig. 30).

#### Psiloderces palopoensis

Taxon classificationAnimaliaAraneaePsilodercidae

Li & Chang sp. nov.

DF4F9334-5917-586E-B62D-FFD2DA799B26

http://zoobank.org/97B946FD-697D-4C94-AFEF-7DE3CA26DB4F

**Figure 14.**
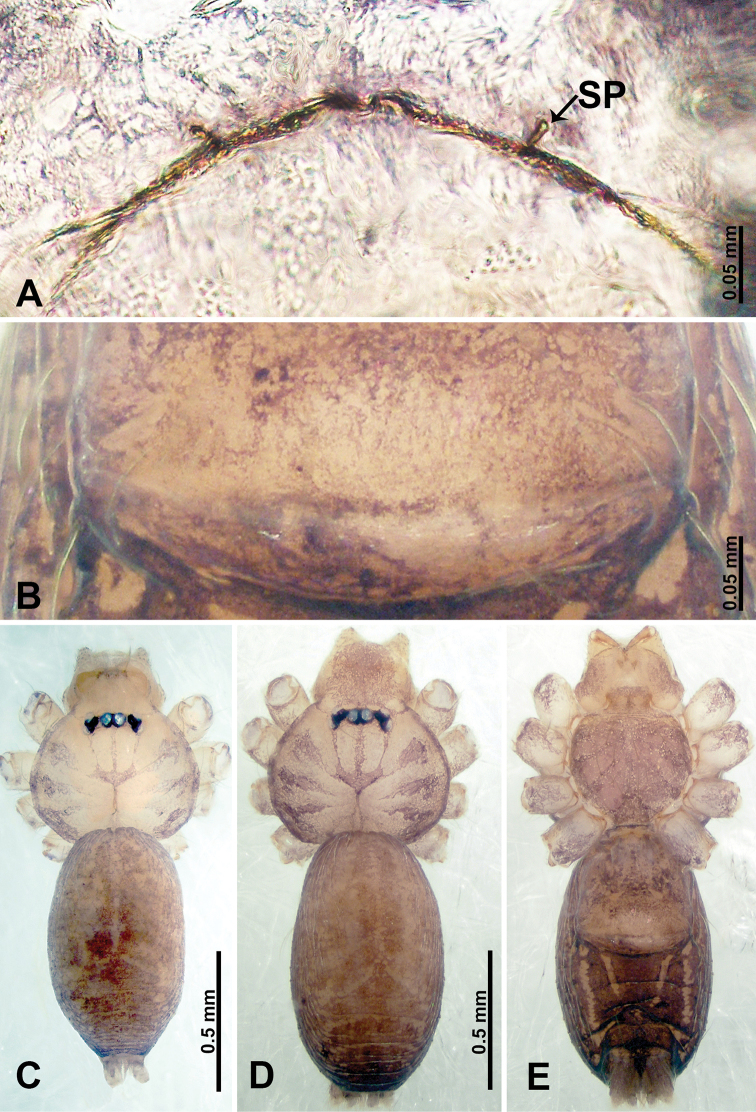
*Psiloderces palopoensis* sp. nov., male holotype and female paratype. **A** Endogyne, dorsal view **B** female epigastric area, ventral view **C** male habitus, dorsal view **D** female habitus, dorsal view **E** female habitus, ventral view. Abbreviation: SP = spermatheca.

**Figure 15.**
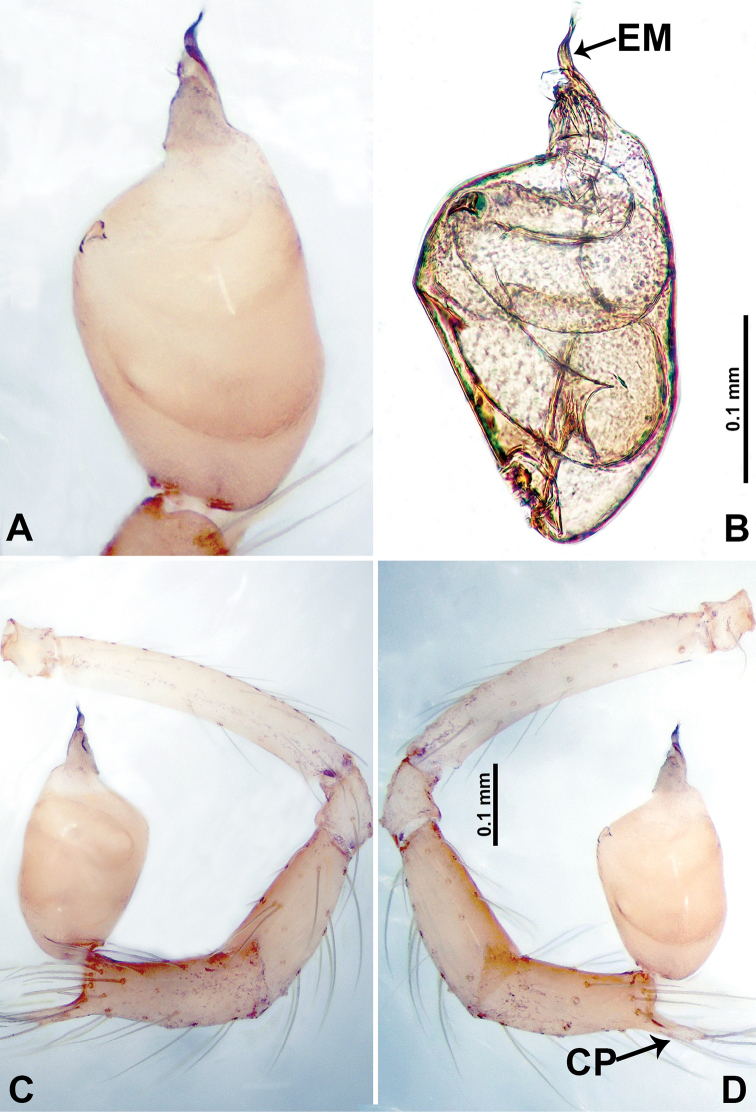
*Psiloderces palopoensis* sp. nov., male holotype. **A** Palp, ventral view **B** bulb, ventral view **C** palp, prolateral view **D** palp, retrolateral view. Abbreviations: CP = cymbial protrusion, EM = embolus.

Figs 14 , 15 , 29C , 30

##### Types.

***Holotype:*** ♂ (IZCAS), Indonesia, Sulawesi, mountain in Palopo, 2°59.9210'S, 120°08.5650'E, 465 m, 2.IX.2017, H. Liu & Z. Chen. ***Paratype***: 1♀ (IZCAS), same data as holotype.

##### Etymology.

The species name is an adjective referring to the type locality.

##### Diagnosis.

Males of *P. palopoensis* sp. nov. can be distinguished from all other species of the genus by the swollen ovate shape of the bulb bearing a short embolus distally (Fig. 15B), 5 times shorter than the length of the tegulum (vs. absence of the extreme length difference of the embolus and tegulum in congeners), and relatively pale color of the male and female; female can be distinguished by widely separated, short, digitiform spermathecae (Fig. 14A).

##### Description.

**Male** (Holotype). Total length 1.41; carapace 0.55 long, 0.63 wide; abdomen 0.86 long, 0.50 wide. Carapace round and pale brown, with trident brown stripes medially and brown patches laterally (Fig. 14C). Chelicerae pale brown (Fig. 29C). Clypeus slanting, purplish. Endites purplish, light brown basally. Labium purplish, delimiting pair of light brown circular dots. Sternum purplish. Abdomen elongated, dorsum with indistinct dark brown pattern posteriorly, antero-ventrally brown with semi-circular patch, posterior with dark brown pattern delimiting light brown dotted vertical lines laterally and V-shaped medially. Legs uniformly brown; measurements: I 5.72 (0.55, 0.16, 1.88, 2.13, 1.00), II 5.78 (1.60, 0.20, 1.60, 1.63, 0.75), III missing, IV 8.33 (2.19, 0.25, 2.60, 2.20, 1.09). Palp (Fig. 15A–D): femur slender, 5 times longer than patella; patella not swollen; tibia 2 times shorter than femur; cymbium almost as wide and long as tibia, with protrusion; bulb pale brown, bulging ovate with embolus arising distally; embolus thinly, sheet-liked, 5 times shorter than entire length of tegulum (Fig. 15B).

**Female** (Paratype). General features and coloration similar to those of male (Fig. 14D, E). Measurements: total length 1.49; carapace 0.55 long, 0.60 wide; abdomen 0.94 long, 0.55 wide. Leg measurements: I 8.27 (2.19, 0.20, 2.50, 2.25, 1.13), II 6.28 (1.75, 0.25, 1.65, 1.75, 0.88), III 4.30 (1.30, 0.20, 1.40, 1.20, 0.20), IV 6.91 (1.88, 0.23, 2.00, 1.80, 1.00). Endogyne (Fig. 14A): widely spaced, digitiform spermathecae, ratio of length of spermatheca and the interdistance of digitiform spermathecae –1:10 (Fig. 14A).

##### Distribution.

Known only from the type locality (Fig. 30).

#### Psiloderces penajamensis

Taxon classificationAnimaliaAraneaePsilodercidae

Li & Chang sp. nov.

D0D5737E-A9A4-5C4B-856E-E41DCEADAE16

http://zoobank.org/82D11755-9750-46AD-A399-281473327D80

**Figure 16.**
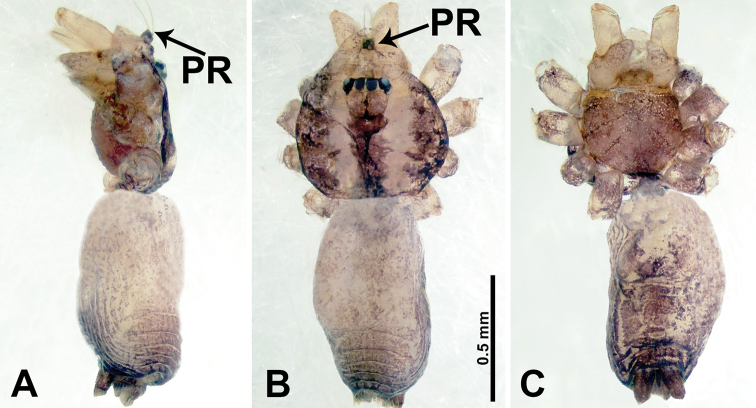
*Psiloderces penajamensis* sp. nov., male holotype. **A** Male habitus, lateral view **B** male habitus, dorsal view **C** male habitus, ventral view. Abbreviation: PR = clypeal projection.

**Figure 17.**
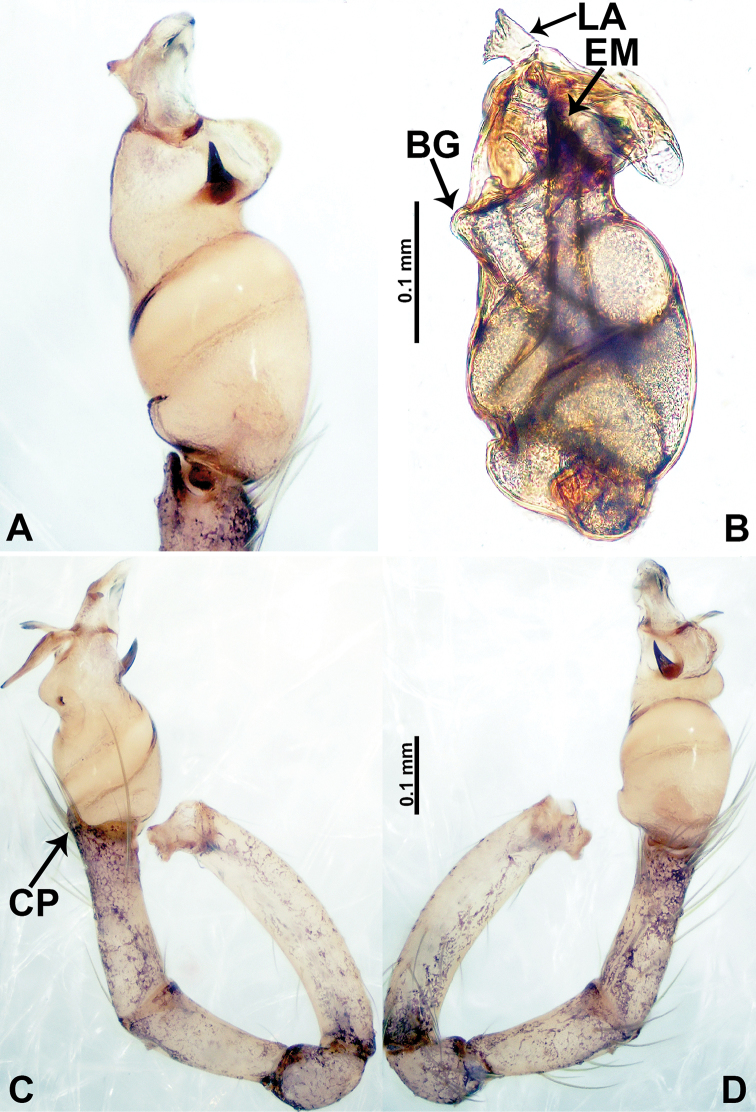
*Psiloderces penajamensis* sp. nov., male holotype. **A** Palp, ventral view **B** bulb, ventral view **C** palp, prolateral view **D** palp, retrolateral view. Abbreviations: CP = cymbial protrusion, BG = bulge, EM = embolus, LA = laminar apophysis.

Figs 16 , 17 , 29A , 30

##### Types.

***Holotype:*** ♂ (IZCAS), Indonesia, East Kalimantan, Penajam, Camp of International Timber Corporation of Indonesia, 1°5.2915'S, 116°41.0938'E, 64 m, 16.VIII.2014, H. Zhao & Z. Yao.

##### Etymology.

The species name is an adjective referring to the type locality.

##### Diagnosis.

The male of *P. penajamensis* sp. nov. can be recognized by the structure of laminar apophysis of the bulb that arises distally, bearing the embolus and bulge (Fig. 17B) (vs. absence of laminar apophysis in congeners), a relatively short clypeal protrusion in which the length does not exceed the anterior tip of the carapace (vs. a clypeal projection that exceeds the anterior tip of carapace).

##### Description.

**Male** (Holotype). Total length 1.20; carapace 0.50 long, 0.60 wide; abdomen 0.70 long, 0.50 wide. Carapace round and brown, with 3 longitudinal dark brown bands, median band and lateral bands nearly the same width (Fig. 16B). Chelicerae brown (Fig. 29A). Clypeus dark brown, bearing few setae, with short medial projection, length does not exceed the anterior edge of carapace, (Fig. 16A, B). Endites brown. Labium dark brown. Sternum dark brown. Abdomen elongated, dorsum with dark brown patches concentrated posteriorly (Fig. 16B), antero-ventrally dark brown with complex patterns (Fig. 16C). Legs uniformly brown; measurements: I–III missing, IV 6.30 (1.56, 0.16, 1.90, 1.80, 0.88). Palp (Fig. 17A–D): femur slender, 3 times longer than patella; patella not swollen; tibia 1.5 times shorter than femur; cymbium almost as long and wide as tibia, protrusion darkens distally; bulb pale yellow, pyriform with bulge, laminar apophysis and embolus arise distally; laminar apophysis sheet-like, almost as long as width of tegulum; laminar apophysis adjacent to embolus, embolus dark short, and pointed; blunt bulge adjacent to embolus (Fig. 17B).

**Female**. Unknown.

##### Distribution.

Known only from the type locality (Fig. 30).

#### Psiloderces grohotensis

Taxon classificationAnimaliaAraneaePsilodercidae

Li & Chang sp. nov.

ABC09172-3C85-5A50-878C-506A7615361B

http://zoobank.org/15F30792-C43A-4D79-AC45-18836A3B24E5

**Figure 18.**
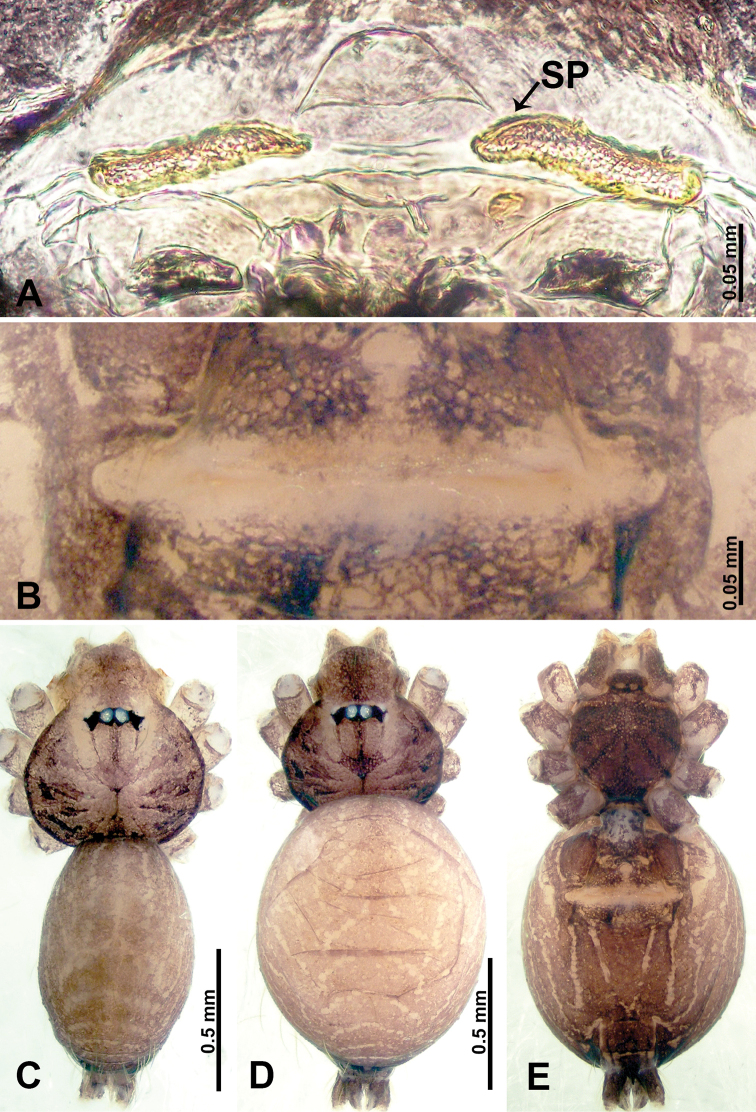
*Psiloderces grohotensis* sp. nov., male holotype and female paratype. **A** Endogyne, dorsal view, **B** female epigastric area, ventral view **C** male habitus, dorsal view **D** female habitus, dorsal view **E** female habitus, ventral view. Abbreviation: SP = spermatheca.

**Figure 19.**
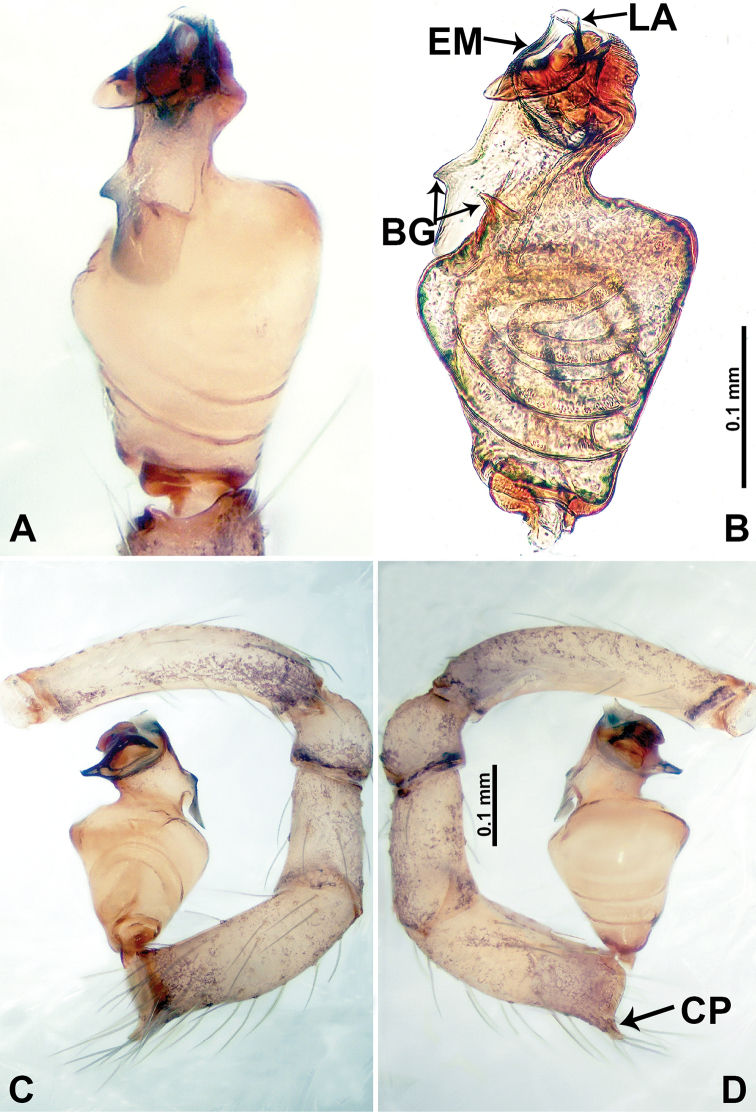
*Psiloderces grohotensis* sp. nov., male holotype. **A** Palp, ventral view **B** bulb, ventral view **C** palp, prolateral view **D** palp, retrolateral view. Abbreviations: CP = cymbial protrusion, BG = bulges, EM = embolus, LA = laminar apophysis.

Figs 18 , 19 , 29D , 30

##### Types.

***Holotype:*** ♂ (IZCAS), Indonesia, East Kalimantan, Tanah Grohot, 1°48.6260'S, 115°51.1250'E, 62 m, 20.VIII.2017, H. Liu & Z. Chen. ***Paratype***: 1♀ (IZCAS), same data as holotype.

##### Etymology.

The species name is an adjective referring to the type locality.

##### Diagnosis.

Males of *P. grohotensis* sp. nov. can be distinguished from all other species of the genus by the structure of the bulb which has 2 bulges, and a laminar apophysis connected to the embolus (Fig. 19B) (vs. one bulge or absent); the female can be distinguished by transversal, tubular spermathecae resembling caterpillars (Fig. 18A) (vs. the absence of transversal, tubular spermathecae).

##### Description.

**Male** (Holotype). Total length 1.30; carapace 0.50 long, 0.60 wide; abdomen 0.80 long, 0.50 wide. Carapace round and pale brown, with trident dark brown stripes medially and dark brown bands laterally (Fig. 18C). Chelicerae pale brown (Fig. 29D). Clypeus slanting, dark brown. Endites dark brown, light brown basally. Labium dark brown, delimiting pair of light brown circular dots. Sternum dark brown. Abdomen elongated, dorsum with indistinct brownish stripes, antero-ventrally with pair of circular dark brown patches followed by elliptical light brown patch, posterior dark brown, delimiting light brown dotted vertical lines laterally and V-shaped medially. Legs uniformly brown; measurements: I 7.47 (2.00, 0.20, 2.25, 2.00, 1.02), II 5.85 (1.60, 0.20, 1.75, 1.50, 0.80), III 4.10 (1.09, 0.16, 1.20, 1.10, 0.55), IV 6.00 (1.60, 0.20, 1.80, 1.60, 0.80). Palp (Fig. 19A–D): femur slender, 4 times longer than patella; patella not swollen; tibia 2.5 times shorter than femur; cymbium 1.5 times shorter than femur, protrusion darkens distally; bulb pale brown, diamond-shaped with embolus and laminar apophysis located distally; embolus thin, connected basally with laminar apophysis; 2 acute bulges directed ventrally (Fig. 19B)

**Female** (Paratype). General features and coloration similar to those of male (Fig. 18D, E). Measurements: total length 1.49; carapace 0.47 long, 0.55 wide; abdomen 1.02 long, 0.78 wide. Leg measurements: I 3.96 (1.30, 0.16, 1.50, 1.30, 0.70), II 4.91 (1.25, 0.16, 1.50, 1.30, 0.70), III 3.89 (1.00, 0.13, 1.13, 1.00, 0.63), IV 5.41 (1.38, 0.13, 1.70, 1.40, 0.80). Endogyne (Fig. 18A): spermathecae tubular, elongate mesally, median tips pointed, lateral ends rounded, receptacles separated by about 3 diameters (Fig. 18A).

##### Distribution.

Known only from the type locality (Fig. 30).

#### Psiloderces bangkiraiensis

Taxon classificationAnimaliaAraneaePsilodercidae

Li & Chang sp. nov.

A5433DD0-0D24-5D43-A239-D0947ABD75AC

http://zoobank.org/A55D77D3-F71B-46B8-ADD8-21259DCDF0EE

**Figure 20.**
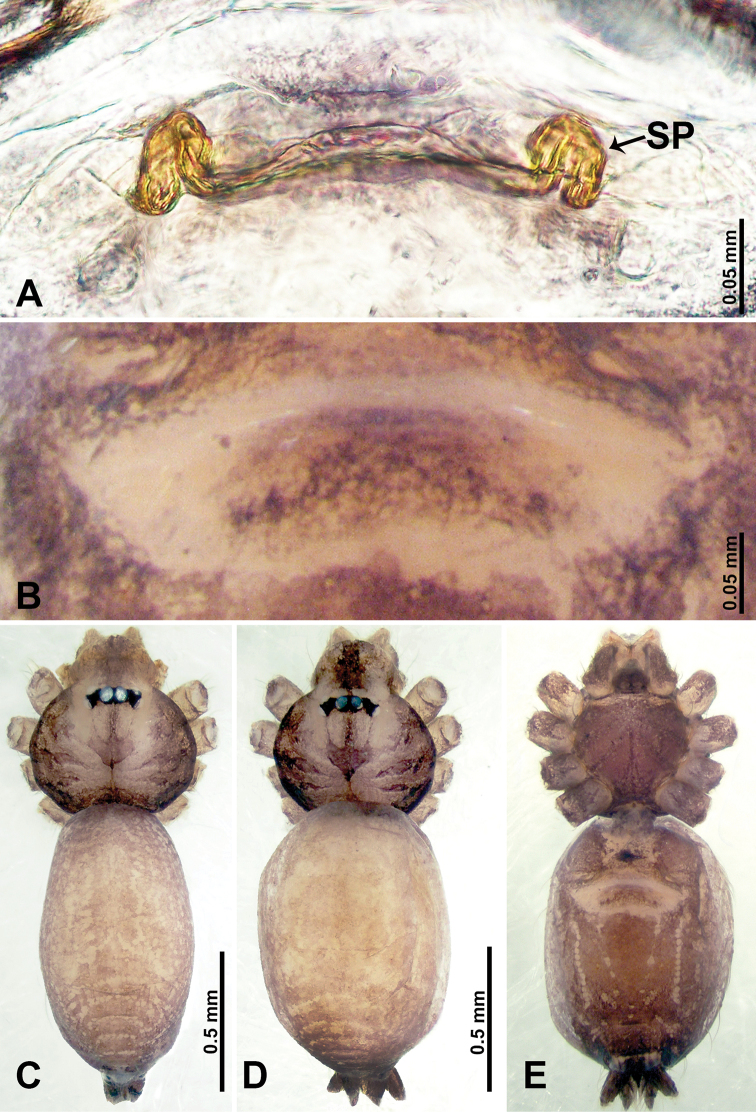
*Psiloderces bangkiraiensis* sp. nov., male holotype and female paratype. **A** Endogyne, dorsal view **B** female epigastric area, ventral view **C** male habitus, dorsal view **D** female habitus, dorsal view **E** female habitus, ventral view. Abbreviation: SP = spermatheca.

**Figure 21.**
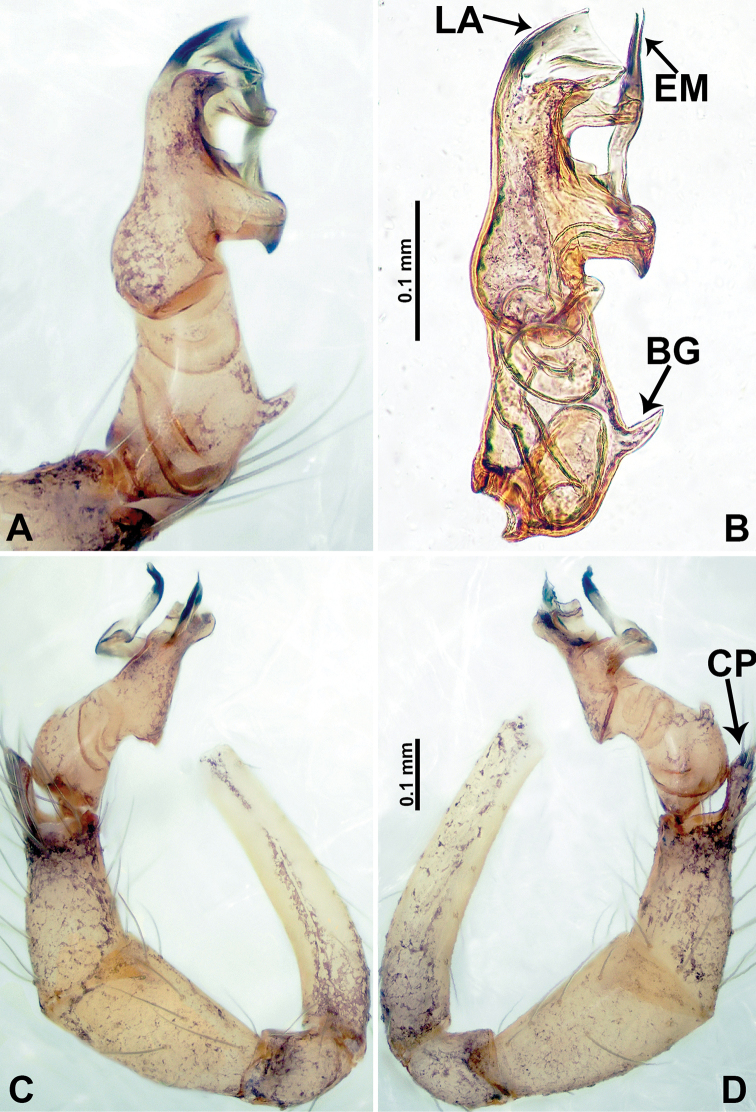
*Psiloderces bangkiraiensis* sp. nov., male holotype. **A** Palp, ventral view **B** bulb, ventral view **C** palp, prolateral view **D** palp, retrolateral view. Abbreviations: CP = cymbial protrusion, BG = bulge, EM = embolus, LA = laminar apophysis.

Figs 20 , 21 , 28A , 30

##### Types.

***Holotype:*** ♂ (IZCAS), Indonesia, East Kalimantan, Kutai Kaetanegara, Bukit Bangkirai, 1°1.2247'S, 116°51.9580'E, 92 m, 18.VIII.2014, Y. Li ***Paratype***: 1♀ (IZCAS), same data as holotype.

##### Etymology.

The species name is an adjective referring to the type locality.

##### Diagnosis.

Males of *P. bangkiraiensis* sp. nov. can be distinguished from all other species of the genus by the relatively slender and elongated bulb bearing a distinct pointed bulge posteriorly (vs. bulb not elongated and slender), the angular tip of the cymbial protrusion (vs. tip of cymbial protrusion rounded), the tibia of the male palp swollen anteriorly (vs. tibia not swollen); females can be distinguished by the curled spermathecae (Fig. 20A).

##### Description.

**Male** (Holotype). Total length 1.49; carapace 0.47 long, 0.60 wide; abdomen 1.02 long, 0.50 wide. Carapace round and brown, with trident dark brown stripes medially and dark brown bands laterally (Fig. 20C). Chelicerae brown (Fig. 28A). Clypeus slanting, dark brown. Endites dark brown, light brown basally. Labium dark brown, delimiting pair of light brown circular dots. Sternum dark brown. Abdomen elongated, dorsum with indistinct brownish stripes posteriorly, with pair of circular dark brown patches antero-ventrally followed by elliptical light brown patch, posterior dark brown pattern delimiting light brown dotted vertical lines laterally. Legs uniformly brown; measurements: I 7.88 (1.88, 0.16, 2.50, 2.34, 1.00), II 5.79 (1.63, 0.16, 1.75, 1.50, 0.75), III 4.41 (1.20, 0.16, 1.25, 1.17, 0.63), IV 6.71 (1.88, 0.20, 2.00, 1.75, 0.88). Palp (Fig. 21A–D): femur slender, 4 times longer than patella; patella not swollen; tibia swollen anteriorly, 1.5 times shorter and 2 times wider than femur; cymbium 2.5 times shorter and 2 times wider than femur, protrusion darkens distally, tip angled; bulb light brown, lanceolate with laminar apophysis and embolus arising distally; laminar apophysis attached to embolus anteriorly and basally; embolus directed apically away from tegulum, thin and slender, bulge directed toward the base of bulb (Fig. 21B).

**Female** (Paratype). General features and coloration similar to those of male (Fig. 20D, E). Measurements: total length 1.38; carapace 0.44 long, 0.55 wide; abdomen 0.94 long, 0.86 wide. Leg measurements: I missing, II missing, III 3.62 (1.00, 0.13, 1.00, 0.94, 0.55), IV 5.51 (1.40, 0.13, 1.72, 1.48, 0.78). Endogyne (Fig. 20A): curled spermathecae connected with bent ducts, equally wide as long (Fig. 20A).

##### Distribution.

Known only from the type locality (Fig. 30).

#### Psiloderces bolang

Taxon classificationAnimaliaAraneaePsilodercidae

Li & Chang sp. nov.

18C20836-25B6-5B4A-8ED6-B7C34434CAE5

http://zoobank.org/A62B1BD7-D14F-4DBF-869F-31938299D18A

**Figure 22.**
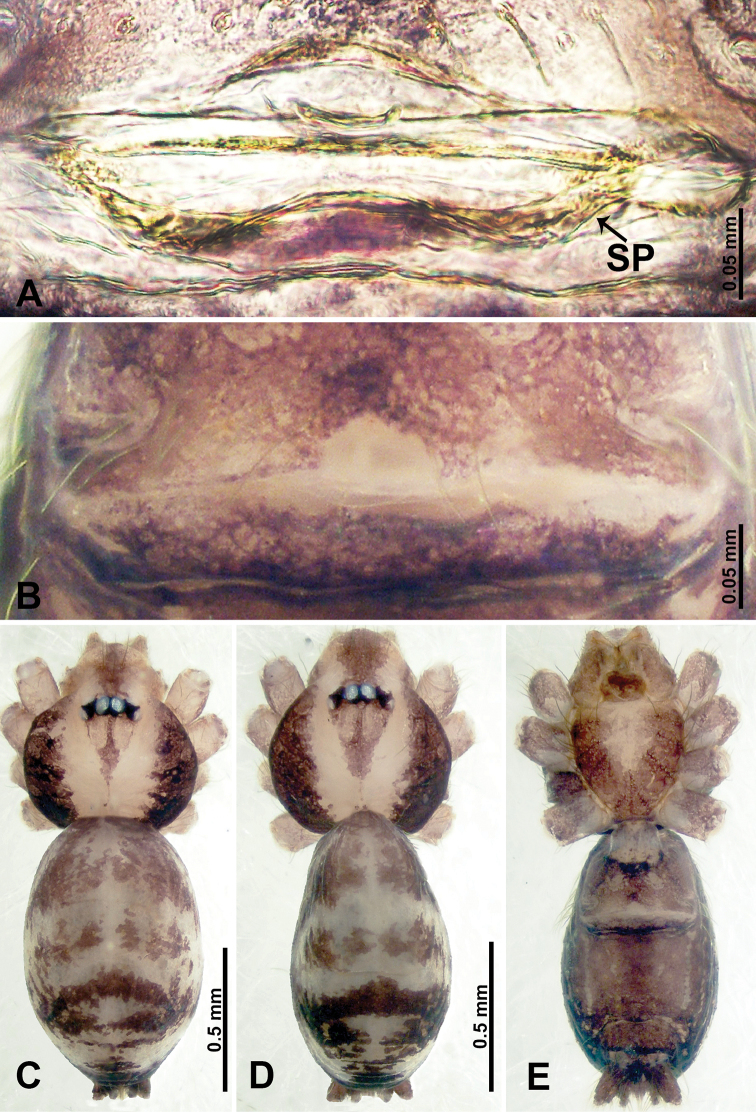
*Psiloderces bolang* sp. nov., male holotype and female paratype. **A** Endogyne, dorsal view **B** female epigastric area, ventral view **C** male habitus, dorsal view **D** female habitus, dorsal view **E** female habitus, ventral view. Abbreviation: SP = spermatheca.

**Figure 23.**
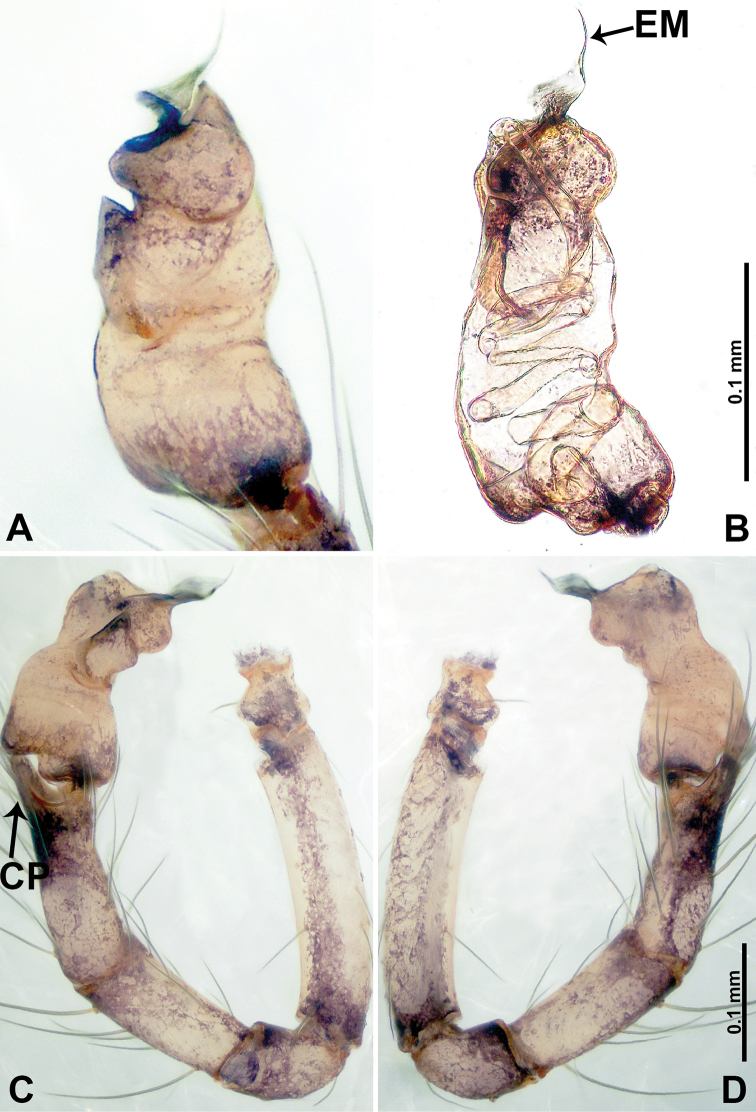
*Psiloderces bolang* sp. nov., male holotype. **A** Palp, ventral view **B** bulb, ventral view **C** palp, prolateral view **D** palp, retrolateral view. Abbreviations: CP = cymbial protrusion, EM = embolus.

Figs 22 , 23 , 29E , 30

##### Types.

***Holotype:*** ♂ (IZCAS), Indonesia, Sulawesi, mountain Palopo, 2°57.6000'S, 120°6.0'E, 509 m, 3.IX.2017, H. Liu & Z. Chen. ***Paratype***: 1♀ (IZCAS), same data as holotype.

##### Etymology.

The species name is a noun in apposition derived from the Chinese pinyin “bōlàng” (wave) and refers to the unique undulated base of the bulb which resembles a wave pattern.

##### Diagnosis.

Males of *P. bolang* sp. nov. resemble those of *P. torajanus* by the trilobate base of the bulb and indented ventrally but can be distinguished by the undivided bulb (vs. bulb distinctly divided into two parts (proximal and distal); figs 8, 9 in Deeleman-Reinhold 1995), the bulb with a crooked periphery and rounded tip (vs. bulb with smooth periphery and pointed tip), the length of cymbial protrusion is at least half the width of the bulb (vs. length of cymbial protrusion narrower than the width of the bulb); females of both species resemble looped spermathecae but can be distinguished by rippled spermathecae (vs. smooth elliptical spermathecae).

##### Description.

**Male** (Holotype). Total length 1.49; carapace 0.47 long, 0.60 wide; abdomen 1.02 long, 0.65 wide. Carapace round and brown, with 3 longitudinal dark brown bands, median band half length the carapace, lateral bands 3 times wider than median band (Fig. 22C). Chelicerae brown, promargin with lamina bearing 2 triangular extensions (Fig. 29E). Clypeus dark brown. Endites dark brown. Labium dark brown delimiting pair of indistinct light brown circular dots. Sternum dark brown, delimiting light brown patch medially. Abdomen elongated, dorsum with dark brown stripes, antero-ventrally with elliptical patch, posterior part with pair of light brown longitudinal lines laterally. Legs uniformly brown; measurements: I 7.73 (2.00, 0.20, 2.40, 2.13, 1.00), II 5.50 (1.30, 0.20, 1.70, 1.50, 0.80), III 5.34 (1.09, 0.16, 1.71, 1.75, 0.63), IV missing. Palp (Fig. 23A–D): femur slender, 3 times longer than patella; patella not swollen; tibia 2 times shorter than femur; cymbium almost as long and wide as tibia, protrusion darkens distally; bulb pale brown, undulate basally and at the margins, pyriform with embolus located anteriorly, with indentation ventrally; embolus laminar and gradually tapering (Fig. 23B).

**Female** (Paratype). General features and coloration similar to those of male (Fig. 22D, E). Measurements: total length 1.30; carapace 0.50 long, 0.60 wide; abdomen 0.80 long, 0.55 wide. Leg measurements: I 4.52 (1.00, 0.16, 1.71, 1.02, 0.63), II missing, III 3.12 (0.80, 0.13, 0.88, 0.81, 0.50), IV 4.60 (1.20, 0.20, 1.40, 1.10, 0.70). Endogyne (Fig. 22A): spermathecae looped transverse anteriorly, rippled posteriorly (Fig. 22A).

##### Distribution.

Known only from the type locality (Fig. 30).

#### Psiloderces wangou

Taxon classificationAnimaliaAraneaePsilodercidae

Li & Chang sp. nov.

D4BE77E9-7D1A-5F4A-B0E0-053F96F5CDAB

http://zoobank.org/696D6C23-F5FC-496D-95BB-30BA6F6EBAEC

**Figure 24.**
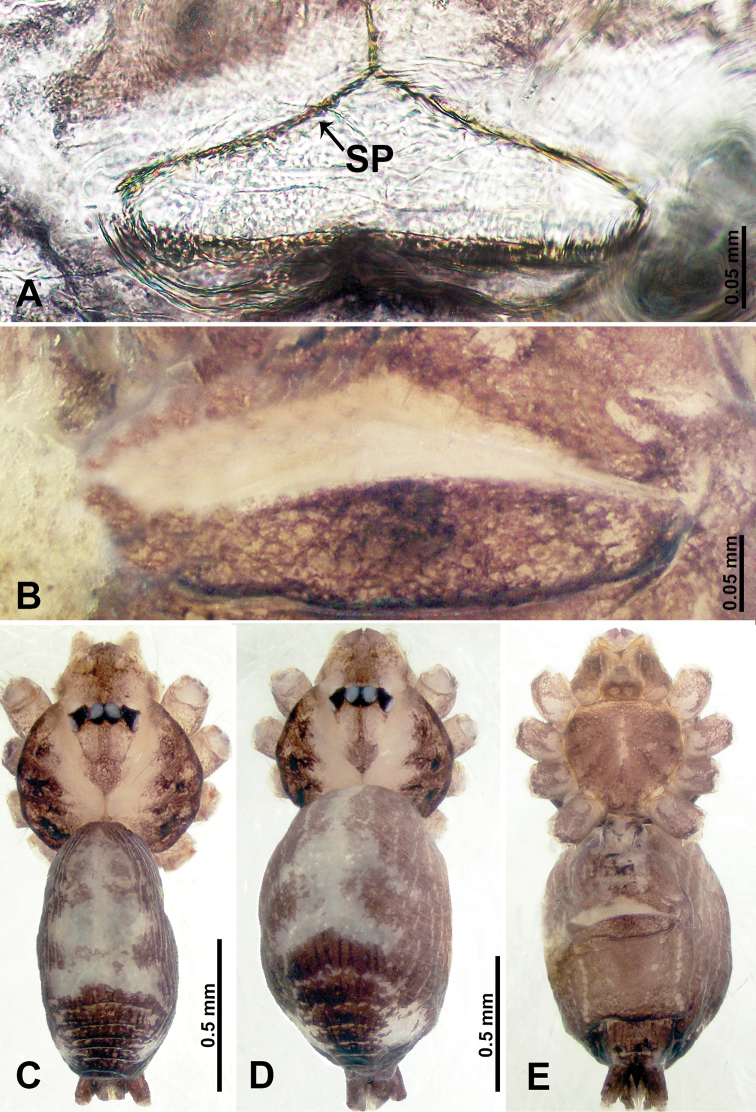
*Psiloderces wangou* sp. nov., male holotype and female paratype. **A** Endogyne, dorsal view **B** female epigastric area, ventral view **C** male habitus, dorsal view **D** female habitus, dorsal view **E** female habitus, ventral view. Abbreviation: SP = spermatheca.

**Figure 25.**
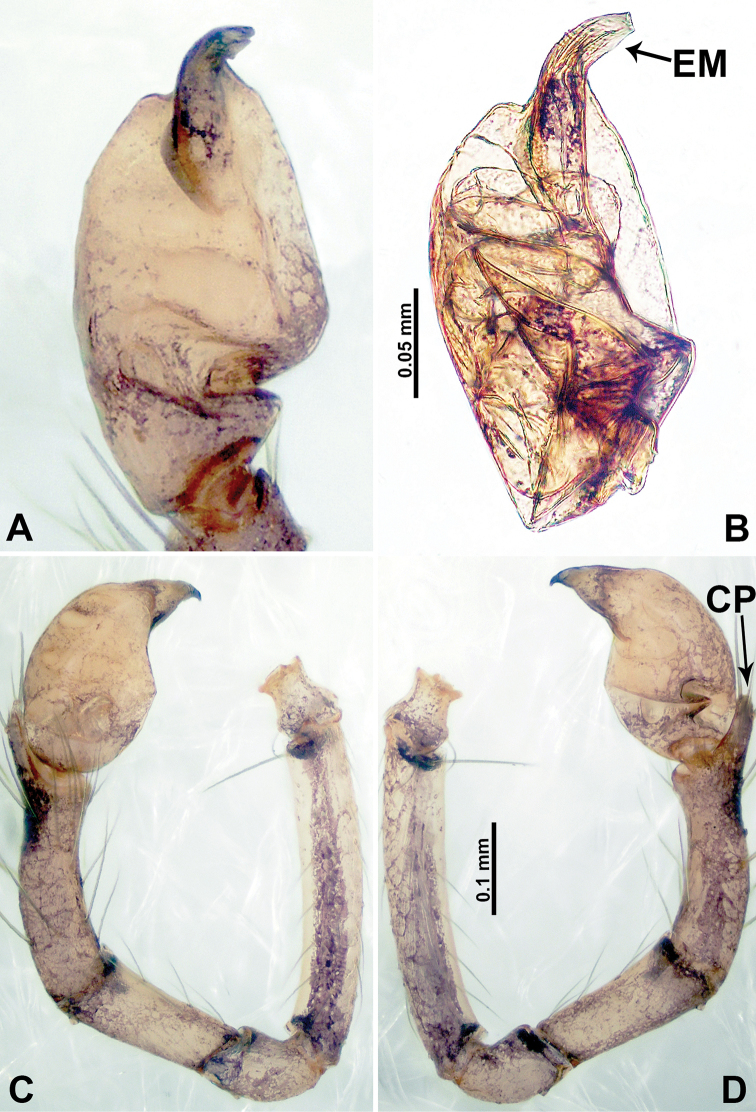
*Psiloderces wangou* sp. nov., male holotype. **A** Palp, ventral view **B** bulb, ventral view **C** palp, prolateral view **D** palp, retrolateral view. Abbreviations: CP = cymbial protrusion, EM = embolus.

Figs 24 , 25 , 28B , 30

##### Types.

***Holotype:*** ♂ (IZCAS), Indonesia, South Sulawesi, Maros, Cenrana Village, East of Maros Water Park, 5°3.2573'S, 119°44.3747'E, 229 m, 24.VII.2014, Y. Li**. *Paratype***: 1♀ (IZCAS), same data as holotype.

##### Etymology.

The species name is a noun in apposition derived from the Chinese pinyin “wāngōu” (hook) and refers to the hook-shaped embolus.

##### Diagnosis.

Males of *P. wangou* sp. nov. resemble those of *P. malinoensis* sp. nov. but can be distinguished by the relatively long, protruding, bent embolus (vs. relatively short and embedded embolus in *P. malinoensis* sp. nov.); females can be distinguished by the enclosed spermathecae (vs. ribbon-like spermathecae with 3 branches).

##### Description.

**Male** (Holotype). Total length 1.30; carapace 0.50 long, 0.56 wide; abdomen 0.80 long, 0.50 wide. Carapace round and brown, with 3 longitudinal dark brown bands, median band half length of carapace, lateral bands 2 times wider than median band (Fig. 24C). Chelicerae brown, promargin with lamina bearing 2 triangular extensions (Fig. 28B). Clypeus dark brown. Endites dark brown, light brown basally. Labium dark brown with pair of light brown circular dots. Sternum dark brown, with light brown median stripe. Abdomen elongated, dorsum with dark brown stripes concentrated posteriorly, antero-ventrally dark brown with elliptical patch, posterior with pair of lateral light brown longitudinal lines. Legs uniformly brown; measurements: I 8.13 (2.19, 0.16, 2.50, 2.19, 1.09), II missing, III 5.04 (1.25, 0.20, 1.25, 1.71, 0.63), IV 6.55 (1.75, 0.20, 2.00, 1.70, 0.90). Palp (Fig. 25A–D): femur slender, 3 times longer than patella; patella not swollen; tibia 2 times shorter than femur; cymbium 1.5 times shorter than femur, protrusion darkens distally; bulb pale brown, pyriform, with embolus arising apically; embolus protruded and slightly bent at tip, resembling a hook, almost 4 times thinner than width of tegulum, length of embolus 2.5 times shorter than tegulum (Fig. 25B).

**Female** (Paratype). General features and coloration similar to those of male (Fig. 24D, E). Measurements: total length 1.50; carapace 0.50 long, 0.63 wide; abdomen 1.00 long, 0.75 wide. Leg measurements: I 6.33 (1.63, 0.20, 2.00, 1.60, 0.90), II missing, III missing, IV 4.90 (1.50, 0.20, 1.70, 0.94, 0.56). Endogyne (Fig. 24A): enclosed spermathecae formed by a horizontal posterior part, and pair of anterior part slanted at 45°, resembling a clothes hanger (Fig. 24A).

##### Distribution.

Known only from the type locality (Fig. 30).

#### Psiloderces malinoensis

Taxon classificationAnimaliaAraneaePsilodercidae

Li & Chang sp. nov.

BDC4F1D2-7DBC-550A-B9E6-3AE21BAA1B39

http://zoobank.org/51DB8482-8B37-4B6D-937C-23358E00E8F5

**Figure 26.**
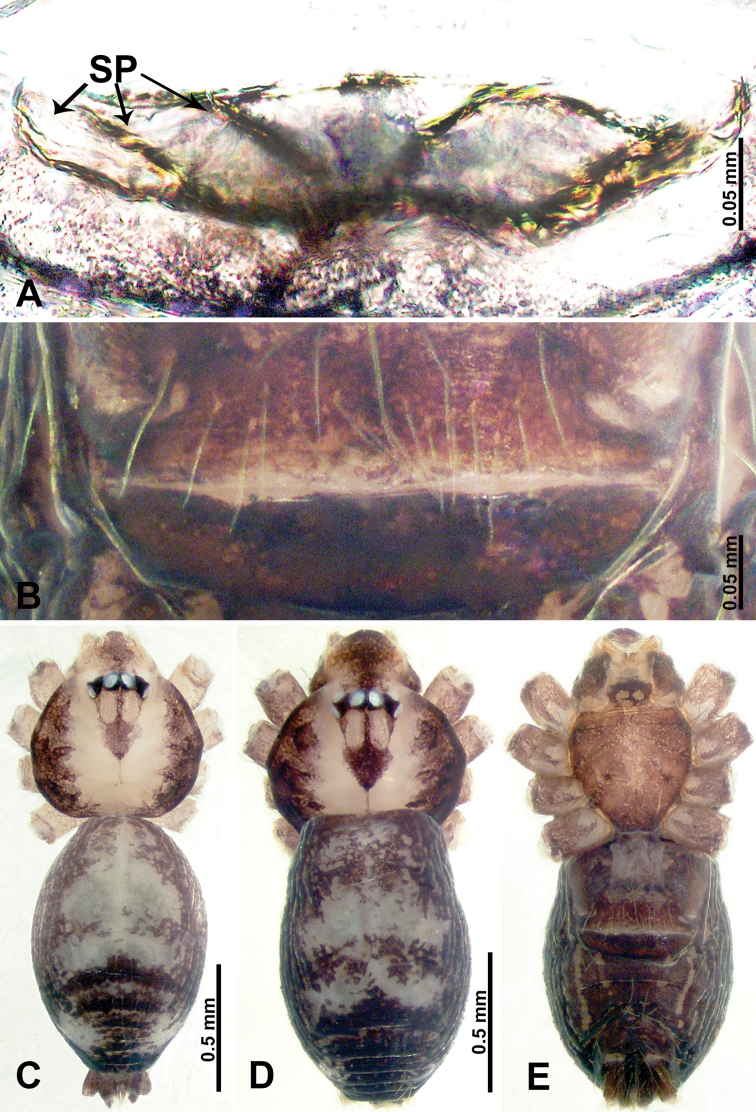
*Psiloderces malinoensis* sp. nov., male holotype and female paratype. **A** Endogyne, dorsal view **B** female epigastric area, ventral view **C** male habitus, dorsal view **D** female habitus, dorsal view **E** female habitus, ventral view. Abbreviation: SP = spermatheca.

**Figure 27.**
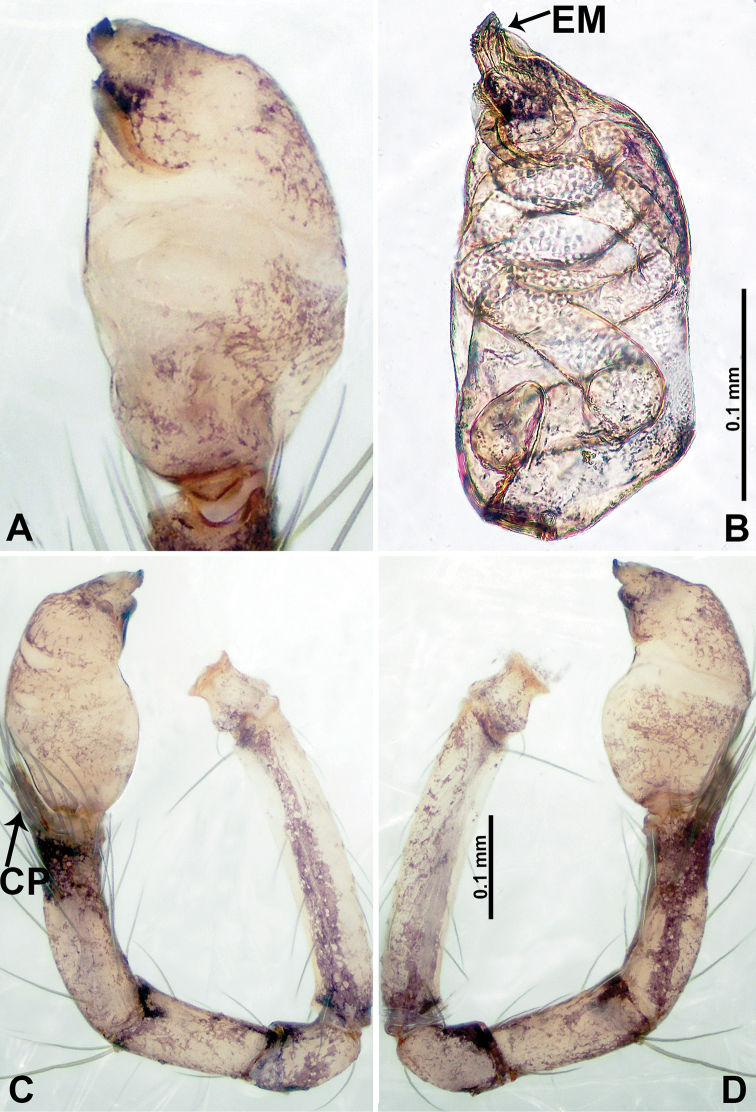
*Psiloderces malinoensis* sp. nov., male holotype. **A** Palp, ventral view **B** bulb, ventral view **C** palp, prolateral view **D** palp, retrolateral view. Abbreviations: CP = cymbial protrusion, EM = embolus.

Figs 26 , 27 , 29B , 30

##### Types.

***Holotype:*** ♂ (IZCAS), Indonesia, Sulawesi, Makassar, mountain around Malino, 5°16.2000'S, 119°50.4000'E, 881 m, 7.IX.2017, H. Liu & Z. Chen. ***Paratype***: 1♀ (IZCAS), same data as holotype.

##### Etymology.

The species name is an adjective referring to the type locality.

##### Diagnosis.

See diagnosis of *P. wangou* sp. nov.

##### Description.

**Male** (Holotype). Total length 1.40; carapace 0.50 long, 0.60 wide; abdomen 0.90 long, 0.63 wide. Carapace round and brown, with trident dark brown stripes medially and dark brown bands laterally (Fig. 26C). Chelicerae brown (Fig. 29B). Clypeus slanting, dark brown medially, light brown laterally. Endites dark brown, light brown basally. Labium dark brown with pair of light brown circular dots. Sternum dark brown. Abdomen elongated, dorsum with dark brown stripes concentrated posteriorly, antero-ventrally with pair of circular dark brown patches followed by semi-circular patch, posterior with light brown dotted vertical lines laterally. Legs uniformly brown; measurements: I 6.64 (1.88, 0.16, 2.00, 1.70, 0.90), II missing, III 4.33 (1.20, 0.16, 1.25, 1.09, 0.63), IV 5.79 (1.50, 0.20, 1.75, 1.48, 0.86). Palp (Fig. 27A–D): femur slender, 4 times longer than patella; patella not swollen; tibia 2.5 times shorter than femur; cymbium 1.5 times shorter than femur, protrusion darkens distally; bulb pale brown, pyriform, with embedded embolus located distally, 4 times shorter than tegulum; tip of embolus pointed (Fig. 27B).

**Figure 28.**
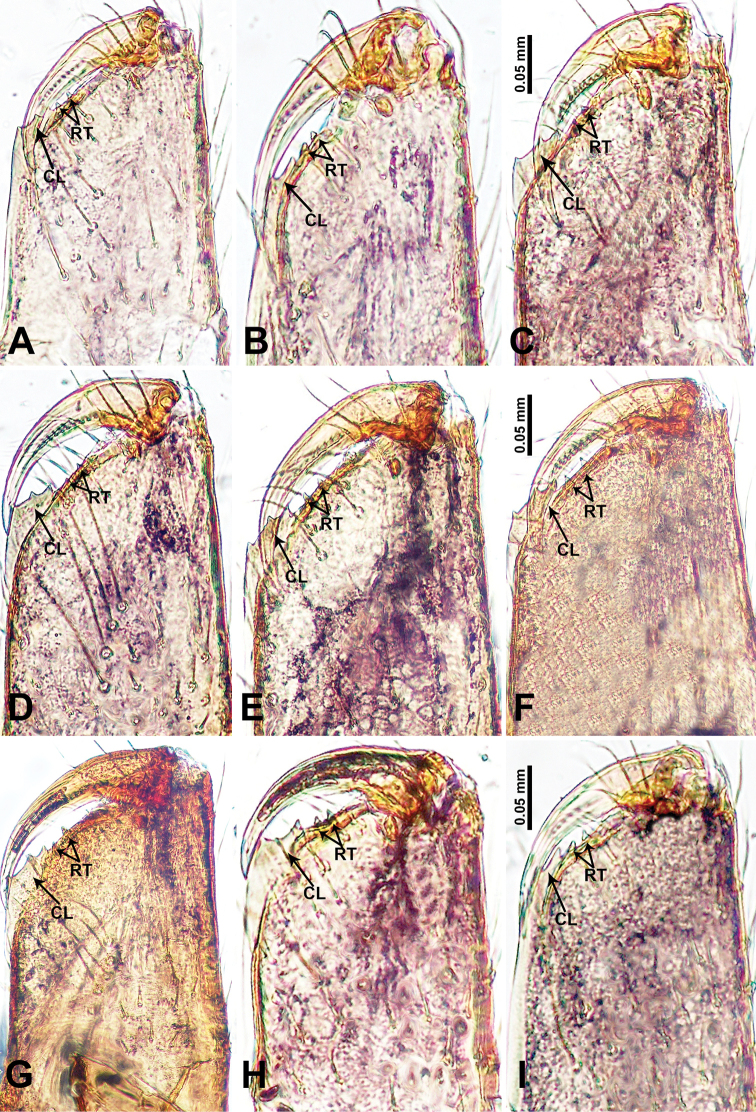
Cheliceral retromargin, posterior view. **A***Psiloderces bangkiraiensis* sp. nov. **B***P. wangou* sp. nov. **C***P. heise* sp. nov. **D***P. cuyapoensis* sp. nov. **E***P. bontocensis* sp. nov. **F***P. gawanaensis* sp. nov. **G***P. xichang* sp. nov. **H***P. cattienensis* sp. nov. **I***P. pingguo* sp. nov. Abbreviations: CL = cheliceral laminar, RT = retromargin teeth.

**Figure 29.**
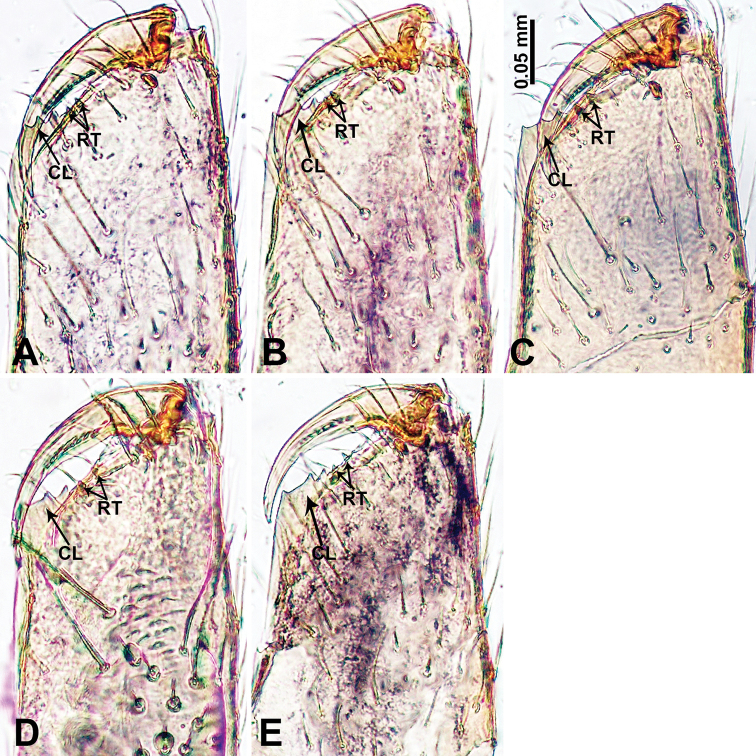
Cheliceral retromargin, posterior view. **A***Psiloderces penajamensis* sp. nov. **B***P. malinoensis* sp. nov. **C***P. palopoensis* sp. nov. **D***P. grohotensis* sp. nov. **E***P. bolang* sp. nov. Abbreviations: CL = cheliceral laminar, RT = retromargin teeth.

**Female** (Paratype). General features and coloration similar to those of male (Fig. 26D, E). Measurements: total length 1.47; carapace 0.47 long, 0.63 wide; abdomen 1.00 long, 0.63 wide. Leg measurements: I 5.63 (1.50, 0.20, 1.75, 1.38, 0.80), II 4.61 (1.25, 0.16, 1.41, 1.09, 0.70), III missing, IV missing. Endogyne (Fig. 26A): ribbon-like spermathecae with 3 branches, lateral pairs longest and directed anteriorly, median and second pairs similar in length, directed laterally (Fig. 26A).

##### Distribution.

**Figure 30.**
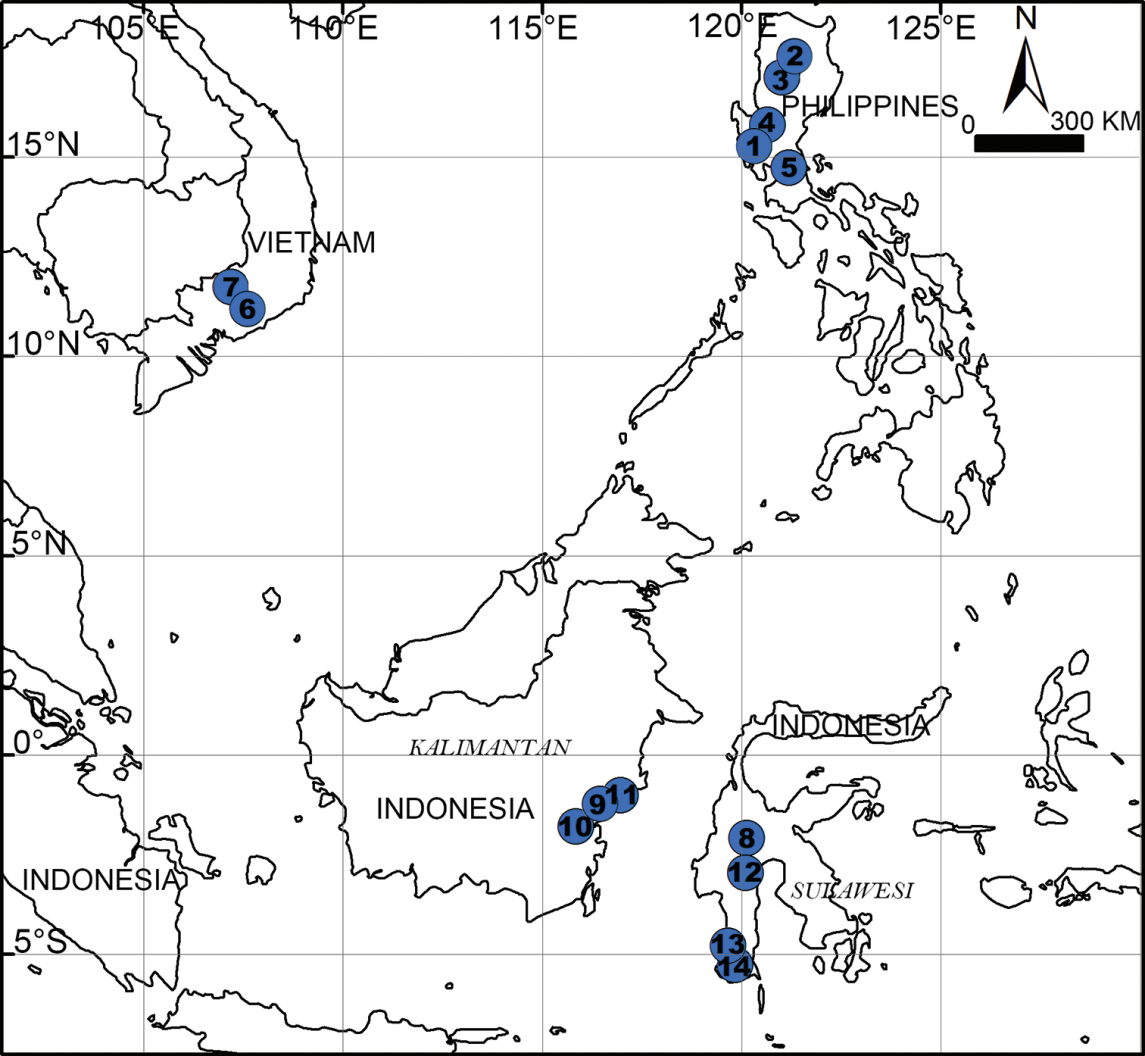
Distribution of new *Psiloderces* species in Southeast Asia. **1***P. heise* sp. nov. **2***P. gawanaensis* sp. nov. **3***P. bontocensis* sp. nov. **4***P. cuyapoensis* sp. nov. **5***P. xichang* sp. nov. **6***P. cattienensis* sp. nov. **7***P. pingguo* sp. nov. **8***P. palopoensis* sp. nov. **9***P. penajamensis* sp. nov. **10***P. grohotensis* sp. nov. **11***P. bangkiraiensis* sp. nov. **12***P. bolang* sp. nov. **13***P. wangou* sp. nov. **14***P. malinoensis* sp. nov.

Known only from the type locality (Fig. 30).

## Supplementary Material

XML Treatment for Psiloderces

XML Treatment for Psiloderces heise

XML Treatment for Psiloderces gawanaensis

XML Treatment for Psiloderces bontocensis

XML Treatment for Psiloderces cuyapoensis

XML Treatment for Psiloderces xichang

XML Treatment for Psiloderces cattienensis

XML Treatment for Psiloderces pingguo

XML Treatment for Psiloderces palopoensis

XML Treatment for Psiloderces penajamensis

XML Treatment for Psiloderces grohotensis

XML Treatment for Psiloderces bangkiraiensis

XML Treatment for Psiloderces bolang

XML Treatment for Psiloderces wangou

XML Treatment for Psiloderces malinoensis
